# Evaluation of Xpert^®^ MTB/RIF Assay in Induced Sputum and Gastric Lavage Samples from Young Children with Suspected Tuberculosis from the MVA85A TB Vaccine Trial

**DOI:** 10.1371/journal.pone.0141623

**Published:** 2015-11-10

**Authors:** Erick Wekesa Bunyasi, Michele Tameris, Hennie Geldenhuys, Bey-Marrie Schmidt, Angelique Kany Kany Luabeya, Humphrey Mulenga, Thomas J. Scriba, Willem A. Hanekom, Hassan Mahomed, Helen McShane, Mark Hatherill

**Affiliations:** 1 South African Tuberculosis Vaccine Initiative, Institute of Infectious Diseases and Molecular Medicine, and Department of Pediatrics and Child Health, University of Cape Town, Cape Town, South Africa; 2 Department of Health, Western Cape Province and Division of Community Health, Stellenbosch University, Stellenbosch, South Africa; 3 Jenner Institute, Nuffield Department of Clinical Medicine, University of Oxford, Oxford, United Kingdom; Faculty of Medicine, AUSTRALIA

## Abstract

**Objective:**

Diagnosis of childhood tuberculosis is limited by the paucibacillary respiratory samples obtained from young children with pulmonary disease. We aimed to compare accuracy of the Xpert^®^ MTB/RIF assay, an automated nucleic acid amplification test, between induced sputum and gastric lavage samples from young children in a tuberculosis endemic setting.

**Methods:**

We analyzed standardized diagnostic data from HIV negative children younger than four years of age who were investigated for tuberculosis disease near Cape Town, South Africa [2009–2012]. Two paired, consecutive induced sputa and early morning gastric lavage samples were obtained from children with suspected tuberculosis. Samples underwent Mycobacterial Growth Indicator Tube [MGIT] culture and Xpert MTB/RIF assay. We compared diagnostic yield across samples using the two-sample test of proportions and McNemar’s χ^2^ test; and Wilson’s score method to calculate sensitivity and specificity.

**Results:**

1,020 children were evaluated for tuberculosis during 1,214 admission episodes. Not all children had 4 samples collected. 57 of 4,463[1.3%] and 26 of 4,606[0.6%] samples tested positive for *Mycobacterium tuberculosis* on MGIT culture and Xpert MTB/RIF assay respectively. 27 of 2,198[1.2%] and 40 of 2,183[1.8%] samples tested positive [on either Xpert MTB/RIF assay or MGIT culture] on induced sputum and gastric lavage samples, respectively. 19/1,028[1.8%] and 33/1,017[3.2%] admission episodes yielded a positive MGIT culture or Xpert MTB/RIF assay from induced sputum and gastric lavage, respectively. Sensitivity of Xpert MTB/RIF assay was 8/30[26.7%; 95% CI: 14.2–44.4] for two induced sputum samples and 7/31[22.6%; 11.4–39.8] [p = 0.711] for two gastric lavage samples. Corresponding specificity was 893/893[100%;99.6–100] and 885/890[99.4%;98.7–99.8] respectively [p = 0.025].

**Conclusion:**

Sensitivity of Xpert MTB/RIF assay was low, compared to MGIT culture, but diagnostic performance of Xpert MTB/RIF did not differ sufficiently between induced sputum and gastric lavage to justify selection of one sampling method over the other, in young children with suspected pulmonary TB.

**Trial Registration:**

ClinicalTrials.gov NCT00953927

## Introduction

Children comprise approximately 6% of the 9 million incident cases of TB globally [1]. Timely, accurate diagnosis of childhood TB across sub-Saharan Africa is hindered by lack of fast, reliable, available diagnostic tests; difficulty in obtaining diagnostic expectorated sputum samples; and the paucibacillary nature of childhood TB disease, particularly when co-infected with HIV [2]. These challenges highlight the importance of obtaining optimal respiratory samples to allow early diagnosis and initiation of therapy for children with TB. The Xpert MTB/RIF assay [Cepheid, Sunnyvale, CA, USA] is an automated nucleic acid amplification test that utilises the GeneXpert® platform to detect *Mycobacterium tuberculosis* and rifampin resistance in less than 2 hours. Sample processing/preparation, PCR amplification and detection are integrated into a single self-enclosed test cartridge, and following sample loading, all steps in the assay are completely automated and self-contained [3,4].

Few studies have compared diagnostic yield of the Xpert MTB/RIF assay between induced sputum and gastric lavage samples, which contain swallowed bacilli originating from the respiratory tract, in young children. Most of these studies are characterized by small sample sizes; wide age band; unmatched or unpaired induced sputum and gastric lavage samples; and include meta-analyses [5–8]. It is important to guide programmatic recommendations on whether increased use of the Xpert MTB/RIF assay should influence choice of sampling method; or whether, for example, Mycobacterial Growth Indicator Tube [MGIT] [Becton Dickinson, Sparks, USA] culture should be preferred over Xpert MTB/RIF for certain samples. Our objective was to compare diagnostic yield of the Xpert MTB/RIF assay on matched induced sputum and gastric lavage samples, obtained from young children with suspected TB in a clinical trial setting, to inform choice of specimen sampling for paediatric TB diagnosis.

## Methods

Data were collected during an infant TB vaccine trial [2009–2012] conducted by the South African Tuberculosis Vaccine Initiative [SATVI] in a rural region about 100 km north of Cape Town, South Africa [9]. The incidence of TB disease in infants was reported as 3% in this study community in 2010 [10]. As previously described [9,11], healthy 4–6 month-old infants were recruited if they had received all age-appropriate routine immunizations, were HIV negative, and if latent TB infection and active TB disease had been excluded. Children were followed quarterly for at least two years to identify signs, symptoms, or household exposure that merited investigation for suspected TB disease i.e.1] weight loss in the preceding two months, 2] cough for more than two weeks without improvement, 3] failure-to-thrive, or 4] conversion to a positive QuantiFERON TB Gold In-Tube test or positive Mantoux test. Symptomatic children were readmitted if they had new symptoms, or if they were not previously treated for TB disease and no alternative diagnosis of their symptoms had been made, or if a previous course of anti-tuberculous treatment had since been completed. Children receiving prophylactic isoniazid monotherapy were also readmitted for investigation if they became symptomatic. Asymptomatic children were admitted for investigation in a dedicated trial specific case verification ward once for each new cohabiting household tuberculosis contact identified, hence the high number of admissions, and readmitted only if they had continued exposure to an inadequately treated cohabiting household tuberculosis contact. Two matched, consecutive induced sputa and early morning gastric lavage samples were obtained from children with suspected TB. Obtained samples underwent Xpert MTB/RIF assay and MGIT culture and drug sensitivity testing [9].

Induced sputum and gastric lavage sample collection have been described previously [9]. Briefly, gastric lavage was performed early in the morning after an overnight fast of at least 4 hours, for each of the first 2 days of admission. Based on age, 5–20 mL of 0.9% saline was instilled and the same volume of gastric lavage fluid aspirated after 2–4 minutes, via a nasogastric tube. The specimen was transported to the lab within 6 hours of collection. Sputum induction was performed 3–4 hours later by a trained professional nurse. The sputum induction procedure was performed in a dedicated procedure room with negative pressure ventilation providing at least 72 air changes per hour by externally vented fan. Standard disinfection and infection control procedures were observed, including use of N95 particulate filter masks by staff. Continuous pulse and oxygen saturation monitoring was used. 100 micrograms of salbutamol was administered prior to the procedure, via a metered dose inhaler with spacer and face mask. Ten minutes after bronchodilator administration, nebulization was performed with 5 mL of sterile hypertonic 5% saline solution, via jet nebulizer attached to cylinder oxygen supply flowing at 3–5 liters/min. Approximately 2.5 mL of induced sputum sample was then obtained by suctioning of the nasopharynx, using a sterile mucus extractor, and transported within 4 hours of collection to the laboratory. Chest physiotherapy was not employed as part of the induced sputum procedure.

Using Xpert MTB/RIF assay and MGIT culture results, we compared diagnostic yield from induced sputum to that from gastric lavage samples; and determined sensitivity of Xpert MTB/RIF assay in each sample type. We defined a TB case by positive result for *Mycobacterium tuberculosis* on any test during a given admission event. For example, an admission event was considered to be a case if there was a positive result on at least one of MGIT culture or Xpert MTB/RIF assay, and not a case if negative on both tests. We report three categories of diagnostic yield: crude yield, incremental yield and differences in yield. Crude yield was defined as number of *Mycobacterium tuberculosis* positive cases divided by number of all admission episodes investigated, for each category of sample. Crude yield was calculated for Xpert MTB/RIF, MGIT culture, and for a combination of the two tests [MGIT culture or Xpert MTB RIF assay]. Incremental yield was defined as the additional gain in crude yield afforded by testing a second sample of induced sputum or gastric lavage, respectively. The numerator for incremental yield was defined as the number of TB cases that might be identified by a specified test, either Xpert MTB/RIF or MGIT culture, or combination of Xpert MTB/RIF and MGIT culture, on a second sample, which were not identified by the first sample. The denominator for incremental yield was the number of admission events with results for two samples of either induced sputum or gastric lavage. For differences in yield, we used the number of admission events positive on either Xpert MTB/RIF assay or MGIT culture on any sample from the same admission event as the denominator. We derived four different numerators for our determination of whether differences in yield were significant. These were 1] admission events positive on Xpert MTB/RIF assay on first induced sputum, 2] admission events positive on Xpert MTB/RIF assay on first gastric lavage sample, 3] admission events positive on Xpert MTB/RIF assay on either first or second induced sputum sample and 4] admission events positive on Xpert MTB/RIF assay on either first or second gastric lavage sample.

Diagnostic yield is reported using proportions and 95% confidence intervals [CI]. We tested whether differences in yield between induced sputum and gastric lavage are significant using the two sample test of proportions. The McNemar’s *χ* ^*2*^ test was used to compare matched observations. Sensitivity and specificity of Xpert MTB/RIF assay was compared between induced sputum and gastric lavage samples, using MGIT culture from all 4 samples during the same admission event as the gold standard. The denominator for sensitivity was the number of TB cases defined by a positive MGIT culture for *Mycobacterium tuberculosis* on at least one of the 4 paired samples [induced sputum or gastric lavage]. The denominator for specificity was the total number of MGIT culture negative admission events. Not all admission events had two gastric lavages and two induced sputa collected and only admission events with complete results were considered in the denominator for specificity. Sensitivity and specificity were calculated using the Wilson’s score method. All statistical tests were two-sided at α of 0.05. Analysis was done in STATA^®^ statistical software version 13.1 for Windows [12].

The clinical trial during which the data were collected received regulatory approval from the University of Cape Town Human Research Ethics Committee; and was registered with the South African National Clinical Trials Register [DOH-27-0109-2654] and ClinicalTrials.gov [NCT00953927] [9]. Parents or legal guardians provided written, informed consent for participation of their children.

## Results

Diagnostic data were available for 1,020 children evaluated for TB disease during 1,214 admissions. Baseline characteristics of all admission events are provided in Table 1. Of 1214 admission events, 693 had one or more of the constitutional symptoms of TB. 44 had weight loss, 196 had cough of more than 2 weeks, 594 had failure to thrive, 430 resided with a household member with a positive sputum smear and 36 had other symptoms. As shown in the supplementary tables (S1 Table and S2 Table), not all admission events had the full complement of 2 induced sputa and 2 gastric lavage aspirate results available for analysis. We used results that gave positive and negative results for *Mycobacterium tuberculosis*. There were 1038 first admissions, 152 second admissions, 23 third admissions and 1 fourth admission.

**Table 1:** Demographic characteristics at baseline of all admission events.

Variable	Placebo arm	Vaccine arm	Overall
**Total admission events[N]**	601[49%]	613[51%]	1214[100%]
**Male**	281[47%]	278[45%]	559[46%]
**Age at admission[Months]**	16.4[11.8;21.3]	17.1[12.4;22.5]	16.8[12.0;22.1]
**Weight at admission[Kgs]**	9.7[8.4–10.8]	10.0[8.6–11.3]	9.9[8.5–11.1]
**Race**			
**Mixed**	528[88%]	528[86%]	1056[87%]
**Black**	72[12%]	84[14%]	156 [13%]
**Asian**	1[<1%]	1[<1%]	2[<1%]
**White**	0[0%]	0[0%]	0[0%]

**Footnote:** Data are N [%] or median [interquartile range]. Kgs = Kilograms.

**Fig 1:**
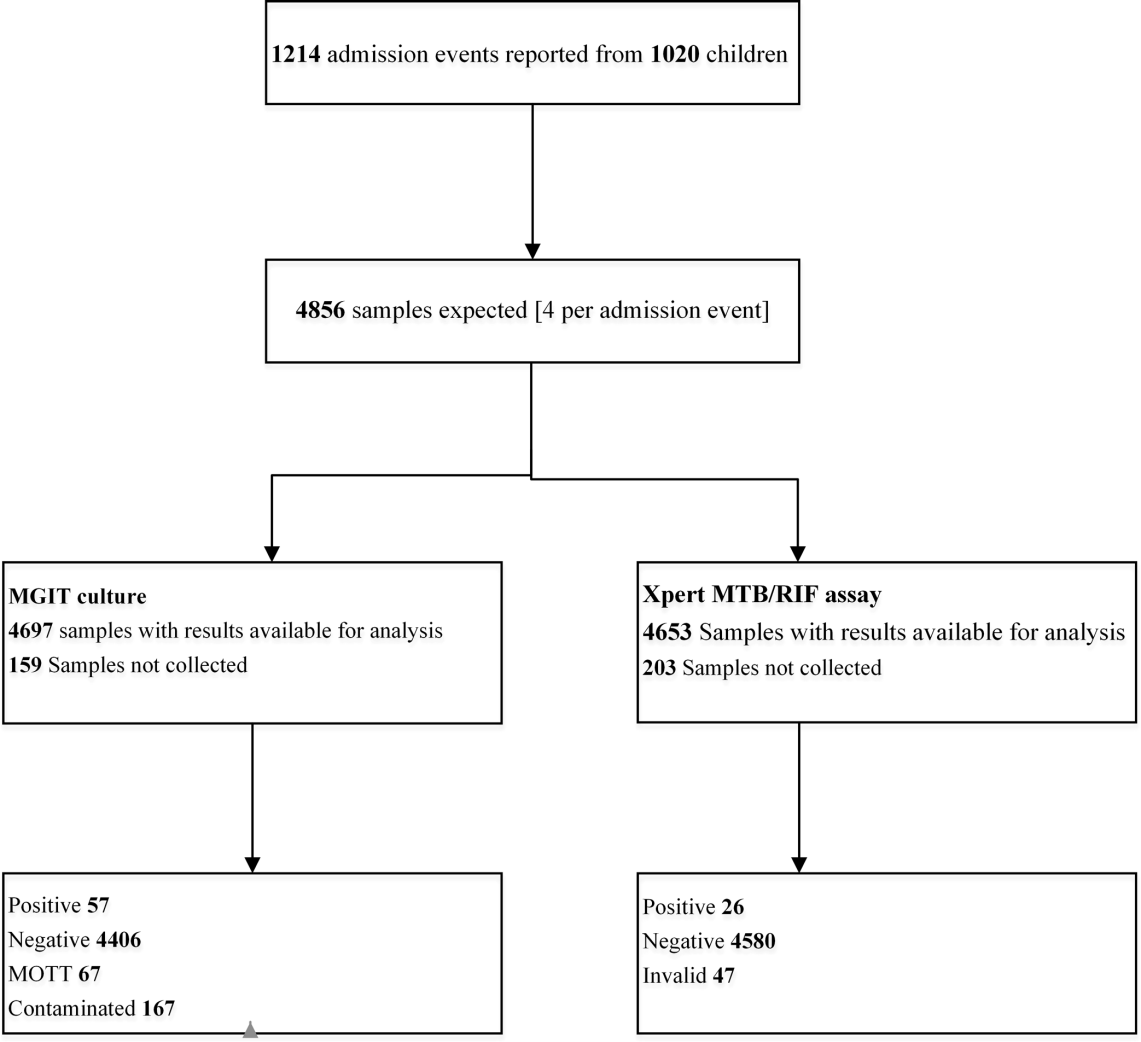
Study schema. Key: MGIT culture = Mycobacterial Growth Indicator Tube Culture; **MOTT** = Mycobacteria other than *Mycobacterium tuberculosis*.

The youngest child diagnosed as a TB case was aged 6.7 months and the oldest 40.5 months; with median age at TB case diagnosis of 21.6 months [IQR: 15.3–29.6]. A total of 57 of 4,463 [1.3%] samples tested positive for *Mycobacterium tuberculosis* on MGIT culture and 26 out of 4,606 [0.6%] samples tested positive on Xpert MTB/RIF assay [Fig 1]. Nineteen children tested positive, on either MGIT culture or Xpert MTB/RIF assay, from the first or second induced sputum sample [n = 19/1028; 2%] and 33 children tested positive, on either MGIT culture or Xpert MTB/RIF assay, from the first or second gastric lavage sample [n = 33/1017; 3%] [Table 2]. There was no statistically significant difference in crude yield of Xpert MTB/RIF between induced sputum and gastric lavage samples.

**Table 2:** Crude yield from induced sputum and gastric lavage samples for *Mycobacterium tuberculosis*.

Sample	Positive Xpert MTB/RIF assay	Positive MGIT culture	Positive MGIT culture or Xpert MTB/RIF assay
**First induced sputum**	n = 7/1,157 [0.6%; 0.3–1.2]	n = 17/1,107 [1.5%; 1.0–2.4]	n = 18/1,093 [1.6%; 1.0–2.6]
**First or second induced sputum**	n = 8/1,128 [0.7%; 0.4–1.4]	n = 19/1,060 [1.8%; 1.2–2.8]	n = 19/1,028 [1.8%; 1.2–2.9]
**Incremental yield by second induced sputum**	n = 1/1,128 [0.1%; 0.0–0.5]	n = 2/1,060 [0.2%; 0.1–0.7]	n = 1/1,028 [0.1%; 0.0–0.5]
**First gastric lavage**	n = 4/1,154 [0.3%; 0.1–0.9]	n = 16/1,094 [1.5%; 0.9–2.4]	n = 18/1,068 [1.7%; 1.1–2.6]
**First or second gastric lavage**	n = 13/1,131 [1.1%; 0.7–2.0]	n = 25/1,048 [2.4%; 1.6–3.5]	n = 33/1,017 [3.2%; 2.3–4.5]
**Incremental yield by second gastric lavage**	n = 9/1,131 [0.8%; 0.4–1.5]	n = 9/1,048 [0.9%; 0.5–1.6]	n = 15/1,017 [1.5%; 0.9–2.4]
**Any induced sputum or gastric lavage**	18/1,103 [1.6%; 1.0–2.6]	36/1,188 [3.0%; 2.2–4.2]	42/1,214 [3.5%; 2.6–4.6]

Data are number of positive admission events, percentage and 95% confidence interval. Numerator is number of admission events with a positive result. Denominator is number of admission events in which the test [or combination of tests] was performed. An admission event was defined as a case if at least one of the test results was positive, and negative if all test results were negative. Not all admission events had two gastric lavages and two induced sputa collected, hence minor variation in the denominator. There were no significant differences in yield of Xpert MTB/RIF assay between induced sputum and gastric lavage samples using our definition of Yield difference Table 3.

**Table 3:** Differences in yield of Xpert MTB/RIF assay test by sample or category of sample.

Specimen	n/N	Yield		Specimen	n/N	Yield	Yield difference	P-value
**First induced sputum**	7/37	**18.9%** [9.5;34.2]	**Vs.**	**First gastric lavage**	4/38	**10.5%** [4.2;24.1]	**8.4%** [-7.6;24.3]	0.304
**First induced sputum**	7/37	**18.9%** [9.5;34.2]	**Vs.**	**Any gastric lavage** ^1^	13/37	**35.1%** [21.8;51.2]	**-16.2%** [-36.1; 3.7]	0.116
**Any induced sputum** ^1^	8/36	**22.2%** [11.7;38.1]	**Vs.**	**First gastric lavage**	4/38	**10.5%** [4.2;24.1]	**-11.7%** [-5.0;28.4]	0.173
**Any induced sputum** ^1^	8/36	**22.2%** [11.7;38.1]	**Vs.**	**Any gastric lavage** ^1^	13/37	**35.1%** [21.8;51.2]	**-12.9%** [-33.4;7.6]	0.223

Data are n/N [95% confidence interval]. Numerator is number of positive results on Xpert MTB/RIF assay only. Denominator is number of positive results on either MGIT culture or Xpert MTB/RIF assay. P-values are not adjusted for multiple comparisons.

^**1**^Numerator was defined as positive Xpert MTB/RIF assay result on at least one of the two samples [i.e. first or second induced sputum, or, first or second gastric lavage]. We observed sensitivity of 23%, 9%, 27% and 23% when Xpert MTB/RIF was performed on one induced sputum, one gastric lavage, two induced sputa and two gastric lavage samples respectively. Diagnostic sampling by induced sputum or gastric lavage did not significantly affect specificity, or positive or negative predictive values of the Xpert MTB/RIF assay, using MGIT culture as the gold standard [Table 4].

**Table 4:** Diagnostic accuracy of Xpert MTB/RIF assay in induced sputum and gastric lavage samples.

Specimen	Sensitivity	Specificity	Positive Predictive Value	Negative Predictive Value
**First induced sputum**	n = 7/31 [**22.6%;** 11.4–39.8]	n = 908/908 [**100%;** 99.6–100]	n = 7/7 [**100%;** 64.6–100]	n = 908/932 [**97.4%;** 96.2–98.3]
**First or second induced sputum** ^1^	n = 8/30 [**26.7%;** 14.2–44.4]	n = 893/893 [**100%;** 99.6–100]	n = 8/8 [**100%;** 67.6–100]	n = 893/915 [**97.6%;** 96.4–98.4]
**First gastric lavage**	n = 3/32 [**9.4%;** 3.2–24.2]	n = 902/903 [**99.9%;** 99.4–100]	n = 3/4 [**75.0%;** 30.1–95.4]	n = 902/931 [**96.9%;** 95.6–97.8]
**First or second gastric lavage** ^1^	n = 7/31 [**22.6%;** 11.4–39.8]	n = 885/890 [**99.4%;** 98.7–99.8]	n = 7/12 [**58.3%;** 32.0–80.7]	n = 885/909 [**97.4%;** 96.1–98.2]

The denominator for sensitivity was any positive MGIT culture on any induced sputum or gastric lavage sample taken during the same admission event. The denominator for specificity was number of admission events with negative MGIT culture on two induced sputa and two gastric lavages [i.e. all 4 results available and negative]. Not all admission events had the full complement of two gastric lavages and two induced sputa collected, or with negative or positive results for inclusion in this analysis. For example, 159 samples were missing/not obtained hence variation in the denominator. See Supplementary S1 and S2 Tables for a full breakdown of results from processing of samples.

^**1**^Xpert MTB/RIF assay was considered positive if at least one of the two test results on the first or second sample was positive.

## Discussion

We report a rigorous, standardized, head-to-head comparison of the diagnostic accuracy of Xpert MTB/RIF assay in induced sputum and gastric lavage samples from more than one thousand young HIV-negative children with suspected TB. Our study suggests that diagnostic performance of Xpert MTB/RIF assay by induced sputum or gastric lavage sampling method is similar, which would support current recommendations by the South African Department of Health [13]. However, we are unable to make a definitive conclusion that there is no difference in performance, due to limited statistical power. We report low sensitivity, moderate-high positive predictive value, high negative predictive value, and very high specificity for Xpert MTB/RIF assay in both sampling techniques.

The relatively low MGIT culture and Xpert MTB/RIF assay positivity rates for *Mycobacterium tuberculosis* that we observed may be explained by the generally mild clinical forms of TB disease in a clinical trial setting with active TB surveillance that generally enabled early case detection and timely referral of children who required isoniazid prophylactic therapy, exclusion of children with co-morbid diseases such as HIV and the young age of the study population. The minimum criteria of admission for investigation to our trial-specific case verification ward was a history of household contact with an individual with pulmonary TB, hence the high number of admissions. These factors have been observed in several studies as potential causes of low rates of culture-positive TB disease in children [8,14–17]. We report lower sensitivity of Xpert MTB/RIF assay in young children than the 62% reported for children aged up to 15 years in a meta-analysis of 15 studies in 2015 [5]. The minimum number of bacilli that can be detected with 95% confidence [i.e. the analytical limit of detection] is 131 colony forming units [CFU] [95% CI: 106–176] per ml of clinical sputum for Xpert MTB/RIF assay [3,4], 10–100 CFU/ ml for MGIT culture [18,19], and 5,000–10,000 CFU/ml for smear microscopy [20]. The paucibacillary nature of samples from young children partly explains the lower sensitivity of Xpert MTB/RIF assay than that observed in adults. Use of a reference standard that takes into consideration results of 4 samples taken over the same admission event increases accuracy of the reference standard, but was found to lead to lower sensitivity, compared to when sensitivity was determined using MGIT culture results from one specimen only. It is hard to determine whether differences in volume of sample obtained using the different specimen collection procedures, i.e. 5–20 ml for gastric lavage based on age and 2.5 ml for induced sputum, and splitting of samples for MGIT culture and Xpert MTB/RIF assay had an effect on results obtained through the two specimen collection approaches in our study. We believe this question can best be answered through a study specifically designed to investigate effect of sample volume by the different specimen collection approaches on yield. The research team had long-standing experience of almost ten years in specimen collection of both induced sputum and gastric lavage samples in trial settings and all staff performing these procedures were re-trained. Thus, sampling differences due to differences in rigor of collection of specimens or a learning curve in collection of either of the two specimens is unlikely to have occurred or significantly impacted our findings.

Chang et al suggested the following factors as plausible reasons for false-positive Xpert MTB/RIF assay results: sub-clinical TB relapse, treatment failure due to multi-drug resistant TB and excretion of residual persistent DNA from dead *Mycobacterium tuberculosis* in patients on TB treatment [8]. In our study, only 2 out of 167 children admitted more than once had a positive Xpert MTB/RIF assay result. They did not have a prior diagnosis of TB and all MGIT cultures and smear microscopy on them were negative. No case of multi-drug resistant TB or TB retreatment was reported in our study. Additionally, the observed specificity of almost 100% [i.e. false positivity rate of <0.006] suggests that false positive Xpert MTB/RIF assay results are unlikely. There are few studies in young children that compare performance of induced sputum and gastric lavage samples using Xpert MTB/RIF assay. For example, Bates *et al* [7] found no significant difference in diagnostic accuracy of Xpert MTB/RIF assay between gastric lavage and sputum. However, this study used expectorated sputum, un-matched samples, and enrolled older children aged up to 15 years, with more severe clinical presentation of TB and multiple co-morbidities, including HIV prevalence of 31% [7]. A 2015 systematic review on diagnostic accuracy of Xpert MTB/RIF assay in children younger than 16 years did not show a significant difference in sensitivity of Xpert MTB/RIF assay with use of induced or expectorated sputum as compared to use of gastric lavage. It reported a sensitivity of 62% [51–73%] and 66% [51–81%] respectively. Besides inclusion of older children as compared to our study, most studies were conducted on inpatients and included both HIV negative and positive children [5].

Our study participants were HIV negative children with mild or moderately severe disease at diagnosis and little comorbidity. This study population differs from that in many sub-Saharan African countries, where HIV and other comorbidities, such as malnutrition, are common and these factors have been shown to influence performance of Xpert MTB/RIF assay [21]. Given the heterogeneity in findings of existing studies comparing performance of Xpert MTB/RIF assay in HIV positive and HIV negative children [7,14,22], it remains unclear how HIV prevalence might affect Xpert MTB/RIF assay performance in induced sputum and gastric lavage samples. Studies to evaluate specimen processing techniques to optimise diagnostic yield of the Xpert MTB/RIF assay, particularly from non-respiratory samples, may further guide recommendations for sample collection in pulmonary and extra-pulmonary TB [23]. The primary limitation of our study was the small number of TB cases observed, despite the large sample size, which may have limited our statistical power to detect small differences in yield and sensitivity of Xpert MTB/RIF assay between induced sputum and gastric lavage samples [24]. A major strength of our study is the matched design, in which two paired induced sputum and two gastric lavage samples were obtained from study participants, using a standardized TB investigation protocol. We also used results of four samples to determine TB disease status, this increases accuracy of the gold standard considering the limited sensitivity of MGIT culture.

In conclusion, we observed relatively low yield of Xpert MTB/RIF assay, compared to MGIT culture, in both induced sputum and gastric lavage aspirate samples in HIV negative young children in a clinical trial setting in a high TB burden country. Our findings suggest Xpert MTB/RIF assay performance is similar between induced sputum and gastric lavage samples. However, our study had limited statistical power to demonstrate small differences between these two sampling methods, given the low diagnostic yield that we observed. Larger comparative studies in this paediatric population would be required to answer this question conclusively.

## Supporting Information

S1 CONSORT Checklist CONSORT 2010 Checklist. (DOC)

S1 Data Data file. (MHT)

S1 File CONSORT flow chart. (DOC)

S1 Protocol Protocol of the vaccine trial. (PDF)

S1 Table A profile or account of each sample result from MGIT culture for *Mycobacterium tuberculosis*. (DOCX)

S2 Table A profile or account of each sample result from Xpert MTB/RIF for *Mycobacterium tuberculosis*. (DOCX)
