# Supplementary material for: Evaluation of Xpert® MTB/RIF Assay in Induced Sputum and Gastric Lavage Samples from Young Children with Suspected Tuberculosis from the MVA85A TB Vaccine Trial
Source: PLoS One. 2015 Nov 10;10(11):e0141623. doi: 10.1371/journal.pone.0141623 (PMC4640848; doi:10.1371/journal.pone.0141623)
Supplement: S1 File — (DOC) [file pone.0141623.s003.doc]

**Figure 1**

**Allocation**

**Analysis**

**Follow-Up**

**Enrollment**

4754 infants consented

1957 excluded

- 25 deaths
- 281 QFT+
- 138 household tuberculosis contact
- 33 HIV exposed
- 947 withdrawal/relocation
- 533 other

Analysed (n=1395)
♦ Excluded from analysis (n=1)

- Dosing deviation

Early discontinuation (n=94)

- 65 loss to follow up
- 25 withdrew consent
- 4 deaths

Allocated to placebo (n=1398)

♦ Received allocated intervention (n=1396)

♦ Did not receive allocated intervention (n=2; 1 ill after randomisation, 1 ineligible after randomisation)

Early discontinuation (n=105)

- 61 loss to follow up
- 37 withdrew consent
- 7 deaths

Allocated to MVA85A (n=1399)

♦ Received allocated intervention (n=1399)

♦ Did not receive allocated intervention (n=0)

Analysed (n=1399)
♦ Excluded from analysis (n=0)

2797 randomized
