# Supplementary material for: Evaluation of Xpert® MTB/RIF Assay in Induced Sputum and Gastric Lavage Samples from Young Children with Suspected Tuberculosis from the MVA85A TB Vaccine Trial
Source: PLoS One. 2015 Nov 10;10(11):e0141623. doi: 10.1371/journal.pone.0141623 (PMC4640848; doi:10.1371/journal.pone.0141623)

THE LANCET

Supplementary appendix

This appendix formed part of the original submission and has been peer reviewed. We post it as supplied by the authors.

Supplement to: Tameris MD, Hatherill M, Landry BS, et al, and the MVA85A 020 Trial Study Team. Safety and efficacy of MVA85A, a new tuberculosis vaccine, in infants previously vaccinated with BCG: a randomised, placebo-controlled phase 2b trial. *Lancet* 2013; published online Feb 4. http://dx.doi.org/10.1016/S0140-6736(13)60177-4.

Appendix

Table 1: Endpoint definition 2
Any of the following numerical categories

1. Isolation of *M tuberculosis* from any site.
2. Identification *of M tuberculosis* by an approved molecular diagnostic technique from any site.
3. Histopathology diagnostic for tuberculosis disease (such as caseating granulomas)
4. Choroidal tubercle diagnosed by an ophthalmologist
5. Miliary pattern on chest X ray in a HIV negative infant
6. Clinical diagnosis of tuberculous meningitis (CSF protein >0.6 g/L and pleocytosis >50/mm^3 with mononuclear cell >50%) **or** features of basal meningeal enhancement and hydrocephalus on head CT.
7. Vertebral spondylosis
8. A single smear/histology specimen positive for auramine positive bacilli from a normally sterile body site.
9. One of each of the following:
   a) Evidence of mycobacterial infection defined as either 1) two acid fast positive smears each from a separate collection morphologically consistent with mycobacteria from either sputum or gastric aspirate that are not found to be non-tuberculous mycobacteria bacteria on culture, or 2) QFT conversion from negative to positive, or 3) Tuberculin skin test **≥10 mm**, or 4) **household contact with AFB smear positive person**
   AND
   b) radiographic findings compatible with tuberculosis defined as at least one of the following identified independently by at least two out of three paediatric radiologists serving on a blinded review panel: calcified Ghon focus, pulmonary cavity, hilar/mediastinal adenopathy, pleural effusion, or airspace opacification
   AND
   c) clinical manifestations compatible with tuberculosis defined as either 1) cough without improvement for longer than two weeks, or 2) weight loss of at least 10% of body weight for at least 2 months, or 3) failure to thrive (crossing at least one entire major centile band downward) for at least 2 months, where the major centile bands are defined as <97th–90th, <90th–75th, <75th–50th, <50th–25th, <25th–10th, and <10th–3rd weight-for-age centiles

* Bold font indicates where different from endpoint 1

Immunology methods
Immunogenicity was evaluated using an *ex-vivo* interferon-γ (IFNγ) ELISPOT assay, together with PBMC and whole blood intracellular cytokine staining (ICS) assays. Fluorescent antibody panels, gating strategies and analysis methodology were previously described (Scriba T et al, EJI 2010). A single pool of 15-mer peptides spanning the Ag85A protein (2μg/mL per peptide) was used in the ex-vivo IFNγ ELISPOT assay and the PBMC and whole blood ICS assay. Medium alone served as negative control and phytohemagglutinin (PHA, Sigma-Aldrich, 10μg/mL) served as positive control.

Table 2. Number of subject with serious adverse events, safety population

Preferred Term	Placebo (n=1396) n (%) (95% CI)	MVA85A (n=1399) n (%) (95% CI)
Subjects with at least one serious adverse event	258 (18·5) (16·5,20·6)	257 (18·4) (16·4,20·5)
Subjects with at least one serious adverse event resulting in or prolonging hospitalization	255 (18·3) (16·3,20·4)	253 (18·1) (16·1,20·2)
Gastroenteritis	88 (6·3)	91 (6·5)
Pneumonia	25 (1·8)	31 (2·2)
Bronchopneumonia	28 (2·0)	27 (1·9)
Lower respiratory tract infection	29 (2·1)	25 (1·8)
Croup infectious	11 (0·8)	8 (0·6)
Febrile convulsion	12 (0·9)	7 (0·5)
Bronchiolitis	8 (0·6)	10 (0·7)
Thermal burn	10 (0·7)	7 (0·5)
Respiratory tract infection	10 (0·7)	6 (0·4)
Urinary tract infection	4 (0·3)	8 (0·6)
Pulmonary tuberculosis	5 (0·4)	6 (0·4)
Tuberculosis	5 (0·4)	5 (0·4)
Upper respiratory tract infection	3 (0·2)	5 (0·4)
Asthma	3 (0·2)	3 (0·2)
Femur fracture	4 (0·3)	2 (0·1)
Kwashiorkor	1 (0·1)	4 (0·3)
Meningitis	2 (0·1)	3 (0·2)
Oral herpes	2 (0·1)	3 (0·2)
Tonsillitis	3 (0·2)	2 (0·1)
Abscess	3 (0·2)	1 (0·1)
Cellulitis	2 (0·1)	2 (0·1)
Impetigo	2 (0·1)	2 (0·1)
Pyrexia	3 (0·2)	1 (0·1)
Accidental exposure	3 (0·2)	0 (0·0)
Bronchospasm	2 (0·1)	1 (0·1)
Convulsion	1 (0·1)	2 (0·1)
Escherichia urinary tract infection	0 (0·0)	3 (0·2)
Phimosis	2 (0·1)	1 (0·1)
Pneumonia viral	2 (0·1)	1 (0·1)
Sepsis	0 (0·0)	3 (0·2)
Vomiting	0 (0·0)	3 (0·2)
Altered state of consciousness	1 (0·1)	1 (0·1)
Bronchitis	2 (0·1)	0 (0·0)
Concussion	1 (0·1)	1 (0·1)
Constipation	1 (0·1)	1 (0·1)
Dysentery	1 (0·1)	1 (0·1)
Eczema	1 (0·1)	1 (0·1)

Preferred Term	Placebo (n=1396) n (%) (95% CI)	MVA85A (n=1399) n (%) (95% CI)
Injury	2 (0·1)	0 (0·0)
Intussusception	2 (0·1)	0 (0·0)
Lobar pneumonia	2 (0·1)	0 (0·0)
Malnutrition	0 (0·0)	2 (0·1)
Measles	0 (0·0)	2 (0·1)
Pneumonia measles	0 (0·0)	2 (0·1)
Stomatitis	1 (0·1)	1 (0·1)
Varicella	1 (0·1)	1 (0·1)
Abnormal faeces	1 (0·1)	0 (0·0)
Accidental drug intake by child	0 (0·0)	1 (0·1)
Acute respiratory distress syndrome	1 (0·1)	0 (0·0)
Aphthous stomatitis	0 (0·0)	1 (0·1)
Apnoeic attack	1 (0·1)	0 (0·0)
Asphyxia	0 (0·0)	1 (0·1)
Balanitis	0 (0·0)	1 (0·1)
Burn infection	1 (0·1)	0 (0·0)
Burns third degree	1 (0·1)	0 (0·0)
Cerebral infarction	0 (0·0)	1 (0·1)
Cryptorchidism	1 (0·1)	0 (0·0)
Dermatitis	1 (0·1)	0 (0·0)
Diarrhoea	0 (0·0)	1 (0·1)
Empyema	0 (0·0)	1 (0·1)
Encephalitis	1 (0·1)	0 (0·0)
Enteritis	0 (0·0)	1 (0·1)
Excoriation	1 (0·1)	0 (0·0)
Eye injury	1 (0·1)	0 (0·0)
Finger amputation	0 (0·0)	1 (0·1)
Gastro-oesophageal reflux disease	1 (0·1)	0 (0·0)
Guillain-Barre syndrome	0 (0·0)	1 (0·1)
HIV test positive	1 (0·1)	0 (0·0)
Head injury	1 (0·1)	0 (0·0)
Hepatitis A	1 (0·1)	0 (0·0)
Hepatosplenomegaly	1 (0·1)	0 (0·0)
Intestinal functional disorder	1 (0·1)	0 (0·0)
Liver abscess	1 (0·1)	0 (0·0)
Lower respiratory tract infection viral	1 (0·1)	0 (0·0)
Meningitis bacterial	0 (0·0)	1 (0·1)
Meningitis tuberculous	1 (0·1)	0 (0·0)
Meningitis viral	0 (0·0)	1 (0·1)
Near drowning	0 (0·0)	1 (0·1)
Obstructive airways disorder	0 (0·0)	1 (0·1)
Pneumonia respiratory syncytial viral	1 (0·1)	0 (0·0)
Pneumonitis	1 (0·1)	0 (0·0)

Preferred Term	Placebo (n=1396) n (%) (95% CI)	MVA85A (n=1399) n (%) (95% CI)
Pneumonitis chemical	0 (0·0)	1 (0·1)
Skin laceration	0 (0·0)	1 (0·1)
Sleep apnoea syndrome	0 (0·0)	1 (0·1)
Status asthmaticus	0 (0·0)	1 (0·1)
Subcutaneous abscess	0 (0·0)	1 (0·1)
Sudden death	0 (0·0)	1 (0·1)
Tibia fracture	0 (0·0)	1 (0·1)

Table 3: Endpoint case distribution by microbiological confirmation, Per protocol population

Parameter	Placebo (n=1395) n (%)	MVA85A (n=1399) n (%)	Total (N=2794) n (%)
Number of subjects meeting TB Case Definition Endpoint 1	**39 (2·8)**	**32 (2·3)**	**71 (2·5)**
Microbiologically confirmed	20 (1·4)	22 (1·6)	42 (1·5)
Not microbiologically confirmed	19 (1·4)	10 (0·7)	29 (1·0)
Number of subjects meeting TB Case Definition Endpoint 2	**52 (3·7)**	**55 (3·9)**	**107 (3·8)**
Microbiologically confirmed	20 (1·4)	22 (1·6)	42 (1·5)
Not microbiologically confirmed	32 (2·3)	33 (2·4)	65 (2·3)
Number of subjects meeting TB Case Definition Endpoint 3	**177 (12·7)**	**196 (14·0)**	**373 (13.4)**
Microbiologically confirmed	19 (1·4)	19 (1·4)	38 (1·4)
Not microbiologically confirmed	158 (11·3)	177 (12·7)	335 (12·0)

Microbiologically confirmed = Culture (+) or GeneXpert (+).

Phase II Double-blinded Randomized Controlled Evaluation of MVA85A/AERAS-485 for Safety, Immunogenicity and Prevention of Tuberculosis in BCG-vaccinated, HIV-negative Infants

Investigational Product: **MVA85A/AERAS-485**

Aeras Protocol Number: **C-020-485 (Oxford TB020)**

Sponsor:

Aeras
1405 Research Boulevard
Rockville, Maryland 20850 USA
Phone: 001-301-547-2900
Fax: 001-301-547-2901

Principal Investigator Agreement:

I, the undersigned, have reviewed this protocol and agree to conduct this protocol in accordance with Good Clinical Practices (ICH-GCP), the ethical principles set forth in the Declaration of Helsinki, and with local regulatory requirements.

Signature Date

Printed Name and Address

TABLE OF CONTENTS

LIST OF ABBREVIATIONS **5**
STUDY ABSTRACT **7**
1 INTRODUCTION **11**
1.1 Background 11
1.2 Description of MVA85A/AERAS-485 Vaccine 11
1.3 Non-clinical Experience with MVA85A/AERAS-485 12
1.4 Clinical Experience with MVA85A/AERAS-485 12
1.5 Rationale 13
2 STUDY OBJECTIVES and DESIGN **13**
2.1 Objectives 13
2.2 Design 14
2.2.1 Rationale for Blinding Procedures 15
3 STUDY PROCEDURES **16**
3.1 Summary Schedule of Subject Evaluations 16
3.2 Subject Selection 21
3.2.1 Recruitment and Informed Consent 21
3.2.2 Screening 22
3.2.3 Subject Eligibility (Inclusion/Exclusion Criteria) 22
3.3 Assignment to Study Group and Randomization 23
3.4 Study Vaccination 24
3.5 On-study Visit Procedures 25
3.5.1 Pre-vaccination and Post-vaccination Monitoring of Subjects 25
3.5.2 Clinical Assessments and Laboratory Tests 25
3.5.3 Immunology Laboratory Evaluations 26
3.5.4 Safety Evaluations 26
3.5.5 Concomitant Medications 27
3.5.6 Diagnosis of Tuberculosis and Case Verification 27
3.5.7 Subject Follow-up and Contact 28
3.5.8 Subject Participation and Loss to Follow-up 28
4 STUDY VACCINES **29**
4.1 Supplies 29
4.2 Accountability 29
4.3 Receipt and Storage 29
4.4 Vaccine Preparation and Dose 30
4.5 Disposition of Unused Supplies 30
5 SAFETY **30**
5.1 Responsibilities for Insuring the Safety of Trial Subjects 30
5.1.1 Principal Investigator 30
5.1.2 Study Sponsor 31
5.1.3 Local Medical Monitor 31
5.1.4 Global Medical Monitor 32

5.1.5 Safety Monitoring Committee 32
5.1.6 Institutional Review Boards and Ethics Committees 33
5.1.7 National Regulatory Authority 33
5.2 Safety Surveillance During the Study 33
5.3 Definition of Adverse Event 33
5.4 Assessing Severity 34
5.5 Assessing Causal Relationship (Relatedness) 35
5.6 Definition of Adverse Reaction 36
5.7 Solicited Adverse Events and Injection Site Reactions 37
5.8 Assessing “Seriousness” and Serious Adverse Events 38
5.9 Assessing Expectedness 38
5.10 Definition of Suspected Unexpected Serious Adverse Reaction (SUSAR) 39
5.11 Reporting of Serious Adverse Events 39
5.12 Other Events Requiring Immediate Reporting 40
5.13 Adverse Event Treatment, Follow-up and Outcome 40
5.14 Long-term Safety Surveillance 41
5.15 Subject Diary and Daily Temperature Monitoring 41

6 STOPPING RULES 42

7 STATISTICAL CONSIDERATIONS 42
7.1 Subject Populations 42
7.2 Demographics and Protocol Compliance 43
7.3 Efficacy Analyses 43
7.3.1 Case definition of tuberculosis 43
7.3.2 Incidence of TB disease 45
7.4 Immunogenicity Analyses 47
7.4.1 Intracellular Cytokine Staining Assay 47
7.4.2 ELISPOT 47
7.4.3 Whole Blood Assay 48
7.4.4 Correlates of Protection 48
7.4.5 QuantiFERON conversion rate 48
7.4.6 Gene Expression 48
7.5 Safety Analyses 48
7.5.1 Adverse Events 49
7.5.2 Other Safety Assessments 49
7.6 Sample Size Considerations 49
7.7 Interim Immunogenicity Review 50
7.8 Safety Reviews 51
7.9 Final Study Report 51
7.10 Computer Methods 51

8 DATA COLLECTION, MONITORING, AND RECORD RETENTION 51

9 HUMAN SUBJECTS 52
9.1 Ethics and Regulatory Considerations 52
9.2 Institutional Review Board or Independent Ethics Committee 52
9.3 Informed Consent 53

10 STUDY COMPLETION **54**

11 PUBLICATIONS **54**

12 CHANGES TO THE PROTOCOL **54**

12.1 List of Changes from Protocol Version 1.0 to Version 2.0 54
12.2 List of Changes from Protocol Version 2.0 to Version 3.0 56
12.3 List of Changes from Protocol Version 3.0 to Version 4.0 59
12.4 List of Changes from Protocol Version 4.0 to Version 5.0 60
12.5 List of Changes from Protocol Version 5.0 to Version 6.0 63
12.6 List of Changes from Protocol Version 6.0 to Version 8.0 66

13 REFERENCES **67**

Appendix A: Subject Diary **68**

Appendix B: Phlebotomy Analysis **72**

Appendix C: Aeras Pediatric Toxicity Table **73**

Appendix D: SAE Reporting Scheme **77**

LIST OF ABBREVIATIONS

AE	Adverse event(s)
ALP	Alkaline Phosphatase
ALT	Alanine aminotransferase
AST	Aspartate aminotransferase
AUC	Area under the curve
BCG	Bacillus Calmette-Guérin
CBC	Complete blood count
CFR	United States Code of Federal Regulations
CPT	Cell Preparation Tube
CRF	Case Report Form(s)
CTA	Clinical Trials Application
dL	Deciliter
ER	Emergency room
FDA	United States Food and Drug Administration
g	Gram
GCP	Good clinical practices
GGT	Gamma-glutamyl-transferase
GMP	Good manufacturing practices
HIV	Human immunodeficiency virus
ICH	International Conference on Harmonization of Technical Requirements for Registration of Pharmaceuticals for Human Use
ICS	Intracellular cytokine staining
ID	Intradermal
IEC	Independent Ethics Committee
IFN-γ	Interferon gamma
IM	Intramuscular
IND	Investigational New Drug Application
IRB	Institutional Review Board
IV	Intravenous
kDa	Kilodalton
L	Liter
MedDRA	Medical Dictionary for Regulatory Activities
µmol	Micromole(s)
mL	Milliliter(s)
mm	Millimeter(s)
mmol	Millimole(s)
MOTT	Mycobacterium other than tuberculosis
Mtb	*Mycobacterium tuberculosis*
PBMC	Peripheral blood mononuclear cell(s)
pfu	plaque-forming unit
PPD	Purified protein derivative
QFT-G	QuantiFERON®-TB Gold test, Cellestis Ltd.
SAE	Serious adverse event(s)
SMC	Safety Monitoring Committee

SolAE	Solicited adverse event(s)
SOP	Standard operating procedure
SST	Serum separator tube
SUSAR	Suspected unexpected serious adverse reaction(s)
TB	Tuberculosis
TNF-α	Tumor necrosis factor alpha
UCT	University of Cape Town
ULN	Upper limit of normal
WBC	White blood cell

STUDY ABSTRACT

TITLE
Phase II Double-blinded Randomized Controlled Evaluation of MVA85A/AERAS-485 for Safety, Immunogenicity and Prevention of Tuberculosis in BCG-Vaccinated, HIV-negative Infants

RATIONALE
The available live tuberculosis vaccine, BCG provides incomplete protection against pulmonary tuberculosis. Due to natural inhibition of a second dose, a BCG revaccination does not provide much additional protection. MVA85A/AERAS-485 presents a tuberculosis antigen in the setting of a live but non-replicating virus vaccine to increase T-cell immunity and thus protection against tuberculosis. MVA85A/AERAS-485 has been administered to over 300 humans without any vaccine-related serious adverse effects and shows evidence of immunogenicity. This study will evaluate the safety and immunogenicity of MVA85A/AERAS-485 in BCG vaccinated infants and has the potential to demonstrate the ability of the vaccine to prevent tuberculosis.

OBJECTIVES
Primary objective
The primary objective of this study is to evaluate the safety profile of MVA85A/AERAS-485 in BCG-Vaccinated, HIV-negative Infants.

Secondary objective
The secondary objectives are to:

- To evaluate the efficacy of the MVA85A/AERAS-485 vaccine compared to controls in prevention of tuberculosis using an endpoint derived from epidemiological cohort surveys in BCG vaccinated infants.

- To evaluate the immunogenicity of the MVA85A/AERAS-485 vaccine compared to controls as described by flow cytometric intracellular cytokine staining of CD4 and CD8 T cells producing any of three cytokines (IFN-γ, TNF-α, and/or IL-2) after stimulation with a peptide pool of mycobacterial peptides.

- To evaluate the immunogenicity of the MVA85A/AERAS-485 vaccine compared to controls as described by the *ex vivo* ELISPOT test used in previous MVA85A/AERAS-485 human trials.

- To evaluate the immunogenicity of the MVA85A/AERAS-485 vaccine compared to controls as described by the University of Cape Town (UCT) whole blood intracellular cytokine assay.

- To discover correlates of protection from tuberculosis in infants vaccinated with MVA85A/AERAS-485.

- To evaluate the QuantiFERON conversion rate at final study assessment in MVA85A/AERAS-485 recipients compared to controls in subjects without a diagnosis of tuberculosis during the trial.

Exploratory objective: To evaluate the innate immune response to MVA85A/AERAS-485 vaccine in a sequentially-enrolled subset of 100 infants using gene expression and transcriptional profile of blood collected at Study Day 1 as analyzed by microarray analysis.

DESIGN

This is a Phase II double-blinded randomized controlled evaluation of safety, immunogenicity and efficacy of MVA85A/AERAS-485 in BCG vaccinated infants without tuberculosis or HIV infection. Infants (126 to 182 days) will receive study vaccine (MVA85A/AERAS-485 or *Candida* skin test antigen control). The study is planned at a single site in South Africa but may be executed at more than one site. Enrollment is expected to be completed in 21 months. Given completion of enrollment in 21 months, all infants will be followed for at least 15 months after the last infant is enrolled into the study. The total duration of follow-up for each infant is at least 15 months and up to 39 months. Infants will be followed for the entire duration both for the development of tuberculosis and serious adverse events.

All infants entered into the study will receive a conjugated pneumococcal vaccine (Prevenar®) with the scheduled DPT immunizations Study Day -84 through Study Day -14. On enrollment to the study, eligible infants will be assigned to a study group starting with Study Group 1 and they will be randomly assigned within a study group to receive either MVA85A/AERAS-485 or *Candida* skin test antigen control as shown below. Infants will be assigned to a safety cohort (Study Group 1), then into one of three immunological assay evaluation groups (Study Groups 2-4), and finally the remainder of infants will be assigned into the correlate of protection cohort (Study Group 5). At least 330 infants will be randomized in Study Group 1, up to 50-60 infants each in Study Groups 2-4, and the remaining infants will be randomized in Study Group 5 such that the total number of infants randomized on the study will be 2784.

Study Group	Cohort Evaluation	MVA85A/ AERAS-485 (ID)	Control *Candida* skin test (ID)	N
1	Safety (labs and adverse events)	165	165	330
2	ELISPOT Immunogenicity	30	30	60
3	ICS Assay Immunogenicity	30	30	60
4	Whole Blood Immunogenicity	30	30	60
5	Correlate of Protection	1137	1137	2274
	Total	1392	1392	2784

The sample sizes were selected because they are judged to be adequate for safety evaluations. A sample size of 1392 MVA85A/AERAS-485 subjects will give a greater than 75% chance of observing an adverse event that has an approximately 1 in a 1000 actual rate of occurrence. A sample size of 165 MVA85A/AERAS-485 subjects in the specialized Safety Cohort (Study Group 1) will provide an 80% chance of observing at least one adverse event which has an approximate 1% actual rate of occurrence. Given a tuberculosis cumulative incidence of 3% over 18 months in the control group, 1392 subjects per study group will provide a 90% chance of detecting a 60% reduction between the treated and control groups based on a two-sided log rank test at a significance level of 0.05.

EVALUATIONS AND FOLLOW-UP

Infants who are randomized at Study Day 0 will be followed up on a regular schedule at Study Day 7 (±2 days), Study Day 28 (± 5 days), Study Day 84 (± 5 days) and every 84 days (± 7 days) thereafter until the end of the study. Specific evaluations at each follow-up will be determined by the study group to which an infant is enrolled. All infants will be followed for adverse events for 28 days after vaccination and for serious adverse events and the development of tuberculosis until the end of the study.

ANALYSIS OF PRIMARY OBJECTIVE

The safety profile of the treatment groups will be described. Listings will be provided for all subjects with serious adverse events (SAEs). All subjects who receive study vaccine will be included in the analysis and presentation of adverse events; additional analyses for the safety cohort will also be provided to evaluate clinical safety laboratory results. Adverse events will be summarized by treatment group, by study cohort, and for all subjects. Adverse events will also be summarized by severity and by relationship to study vaccine, by treatment group, study cohort, and for all subjects. Adverse events and clinically relevant laboratory results for the safety cohort will be summarized to examine the relationship between treatment group and key safety endpoints including number (percentage) of solicited and spontaneous adverse events, rates of reactogenicity, and number (percentage) of subjects with newly abnormal post-vaccination laboratory values based on predefined neonatal toxicity criteria.

ANALYSIS OF SECONDARY OBJECTIVES

The number (percentage) of subjects with a diagnosis of tuberculosis based on clinically-derived TB diagnostic criteria will be summarized by treatment group, study cohort, and for all subjects. The cumulative person-years incidence of TB and associated 95% confidence intervals during study follow-up will be summarized by treatment group. Comparison of the incidence of TB in each treatment group will be performed using relative risk summaries and corresponding 95% confidence intervals, as appropriate. Time from Study Day 0 to initial TB diagnosis in each treatment group will be examined using the Kaplan-Meier estimate of the survival function; time to diagnosis between the two treatment groups will be explored using the log-rank test or similar methodology based on distributional assumptions.

The immune response of MVA85A/AERAS-485 will be described for subjects in the three immunogenicity cohorts using the *ex vivo* Elispot assay, the whole blood intracellular cytokine assay cohort, or the ICS assay. Response to each assay will be presented at each post-immunization time point, by treatment group, and by study cohort. ICS assay immune response will be expressed as the percentage of CD4 and CD8 T cells producing any one of three cytokines (IFN-γ, TNF-α, or IL-2) or any combination of the three cytokines simultaneously after stimulation with a peptide pool of mycobacterial peptides. Immune responses to MVA85A/AERAS-485 will be compared with control using a linear mixed effects model or other regression models as appropriate. Separate models will be prepared for cytokine responses specific to each assay. Data will be transformed prior to analysis as appropriate.

All infants will have PBMC and plasma collected and stored for future studies of MVA85A/AERAS-485-induced immune correlates of protection. The vaccine-induced immune correlates of protection against tuberculosis are not known. We will compare immune responses between infants who developed tuberculosis (n=60-80, estimated) and those who do not develop tuberculosis.

The number (percentage) of QuantiFERON conversions in all study cohorts who do not receive a diagnosis of TB during the study will be summarized by treatment group, study cohort, and for all subjects.

Innate immune response to vaccination will be examined as an exploratory objective in a sequentially-enrolled subset of 100 randomized subjects. Gene expression and transcriptional profiles of blood collected at Study Day 1 post-vaccination will be analyzed using microarray analysis.

1 INTRODUCTION

1.1 Background

The only currently available tuberculosis vaccine, Bacillus Calmette-Guérin (BCG), is estimated to reduce the risk of tuberculosis in children by about 80% (1). While effective chemotherapy is available, the uncertainties in diagnosis within the affected populations and the expense and toxicity of chemotherapy permit continued morbidity and death. An improved vaccination would have a positive effect on the health of many people.

Heterologous 'prime–boost immunization' strategies involve administration of two different vaccines that deliver the same antigen weeks or months apart. This approach has been demonstrated to elicit higher cellular immunity than a single BCG vaccination in several animal disease models (2,3). Given the protective efficacy of BCG in childhood, BCG would ideally be the priming immunization in such a prime–boost strategy.

The University of Oxford has developed a vaccination strategy with BCG as the priming vaccination and MVA85A/AERAS-485 as the boost. MVA85A/AERAS-485 is a recombinant MVA expressing the *Mycobacterium tuberculosis* antigen Ag85A. MVA is modified vaccinia virus Ankara, a highly attenuated strain of vaccinia virus which does not replicate in mammalian cells. Ag85A is an immunodominant secreted antigen that is highly conserved throughout all mycobacterial species. Importantly for BCG priming, Ag85A is present in all strains of BCG and in all strains of environmental mycobacteria sequenced to date (4).

1.2 Description of MVA85A/AERAS-485 Vaccine

The MVA85A/AERAS-485 vaccine consists of the attenuated vaccinia virus MVA vector with a 1176 base-pair insert, which is almost the complete tuberculosis gene for Ag85A, with the tissue plasminogen activator (TPA) signal sequence preceding the N terminus and a monoclonal antibody tag (pk) at the C terminus. Expression of the tuberculosis DNA sequence is regulated by the vaccinia P7.5 early/late promoter.

Ag85A is a secreted 32-kDa protein mycobacterial antigen that is found in abundance in culture fluids of *Mycobacterium tuberculosis* (*Mtb*). It is a member of mycolyl transferase complex and is highly immunogenic. It contains several CD4 T cell epitopes and at least one CD8 T cell epitope and protects against *Mtb* challenge in both mice and guinea pigs (5,6). It is known that BCG-vaccinated mice lose immunity against challenge infection as they age. Brooks and colleagues showed that when these animals were vaccinated with the Ag85A in midlife, they regained protection in the lungs comparable to those of young mice with minimal pathological damage. Immunization of inbred mice with DNA encoding the Ag85A induced statistically significant protective immunity against challenge with *Mtb*. Protection was assessed by measuring the amount of live *Mtb* in the lungs (7). The magnitude of protection with the DNA vaccine was 1–1.5 logs more than placebo-vaccinated animals and similar to BCG vaccine. DNA-Ag85A vaccine induced less protection than BCG in a highly-susceptible guinea pig model (8). However, the prime–boost immunization in mice involving

MVA expressing Ag85A resulted in both CD4+ and class I-restricted CD8+ T cell responses and protected these animals against TB. The protection was equivalent to that of traditional BCG vaccination (6). Furthermore, MVA85A/AERAS-485in humans significantly boosted BCG-primed anti-TB immunity after it was given to 31 adult volunteers and produced levels of antigen-specific IFN-γ-secreting T cells 5–30 times higher than in people given a BCG vaccination (9). Ag85A is an important protective antigen.

1.3 Non-clinical Experience with MVA85A/AERAS-485

Protective efficacy studies using a BCG prime-MVA85A/AERAS-485 boost strategy, followed by *Mtb* challenge, have been conducted in 4 animal species (mice, guinea pigs, non-human primates and cattle). These experiments suggested improved protection of the combination compared to BCG alone.

The toxicity of MVA85A/AERAS-485 was tested in mice following 2 ID injections of 1×10^7 pfu MVA85A/AERAS-485 over 15 days. Control animals received PBS. The mice were sacrificed 14 days after the second dose. MVA85A/AERAS-485-injected mice showed no differences from PBS-injected control animals in body weight, organ weight, histopathology, or haematological and biochemical parameters. A low level of local irritation at the site of administration was observed with MVA85A/AERAS-485.

1.4 Clinical Experience with MVA85A/AERAS-485

A total of 13 clinical studies (11 Phase I, 2 Phase II) of MVA85A/AERAS-485 have been completed or are ongoing. A total of 102 healthy adults have received MVA85A/AERAS-485 in the completed studies, and 200 subjects (66 adults, 12 adolescents, 24 children, 98 infants) have been vaccinated in the ongoing studies. The doses of MVA85A/AERAS-485 in these studies range from 1×10^7 to 1×10^8 pfu.

MVA85A/AERAS-485 induced high levels of antigen-specific IFN-γ secreting T cells in BCG-naïve adults, and even higher levels in adults previously vaccinated with BCG either 1 month previously or 1-20 years previously. Immunogenicity of MVA85A/AERAS-485 in BCG-vaccinated adolescents and adults latently infected with *Mtb* was similar to that seen for BCG-vaccinated healthy adults. In BCG-vaccinated infants, induction of measurable levels of antigen-specific T cells was observed, with similar results seen when MVA85A/AERAS-485 was given either on its own or concomitantly with EPI vaccines.

Over 300 individuals have received MVA85A/AERAS-485 in clinical trials to date, including BCG-naïve adults; BCG-vaccinated adults, adolescents, children, and infants; HIV positive adults and adults latently infected with *Mtb*. Final safety data are available for 129 of these subjects. No serious adverse events judged to be related to MVA85A/AERAS-485 have been reported in these subjects. The safety profile of MVA85A/AERAS-485 developed so far consists of local adverse events in most subjects. Although there were some severe local reactions such as erythema, swelling, and pain at the injection site, most local reactions were typically mild in severity and most resolved within 1 week of vaccination. Less frequent systemic reactions included constitutional manifestations resembling flu such as fever, headache, rash, malaise, fatigue, anorexia, cough, arthralgia, and myalgia. These systemic

events were also usually mild and all spontaneously resolved within 24-48 hours after vaccination.

1.5 Rationale

The available live tuberculosis vaccine, BCG provides incomplete protection against pulmonary tuberculosis. Due to natural inhibition of a second dose, a BCG revaccination does not provide much additional protection. MVA85A/AERAS-485 presents a tuberculosis antigen in the setting of a live but non-replicating virus vaccine to increase T-cell immunity and thus protection against tuberculosis. MVA85A/AERAS-485 has been administered to over 300 humans without any vaccine-related serious adverse effects and shows evidence of immunogenicity. This study will evaluate the safety and immunogenicity of MVA85A/AERAS-485 in BCG vaccinated infants and has the potential to demonstrate the ability of the vaccine to prevent tuberculosis.

2 STUDY OBJECTIVES and DESIGN

2.1 Objectives

Primary objective

The primary objective of this study is to evaluate the safety profile of MVA85A/AERAS-485 in BCG-vaccinated, HIV-negative infants.

Secondary objective

The secondary objectives are:

- To evaluate the efficacy of the MVA85A/AERAS-485 vaccine compared to controls in prevention of tuberculosis using an endpoint derived from epidemiological cohort surveys in BCG vaccinated infants.

- To evaluate the immunogenicity of the MVA85A/AERAS-485 vaccine compared to controls as described by flow cytometric intracellular cytokine staining of CD4 and CD8 T cells producing any of three cytokines (IFN-γ, TNF-α, and/or IL-2) after stimulation with a peptide pool of mycobacterial peptides.

- To evaluate the immunogenicity of the MVA85A/AERAS-485 vaccine compared to controls as described by the *ex vivo* ELISPOT test used in previous MVA85A/AERAS-485 human trials.

- To evaluate the immunogenicity of the MVA85A/AERAS-485 vaccine compared to controls as described by the UCT whole blood intracellular cytokine assay.

- To discover correlates of protection from tuberculosis in infants vaccinated with MVA85A/AERAS-485.

- To evaluate the QuantiFERON conversion rate at final study assessment in MVA85A/AERAS-485 recipients compared to controls, in infants without a diagnosis of tuberculosis during the trial.

Exploratory objective: To evaluate the innate immune response to MVA85A/AERAS-485 vaccine in a sequentially-enrolled subset of 100 infants using gene expression and transcriptional profile of blood collected at Study Day 1 as analyzed by microarray analysis.

2.2 Design

This is a Phase II double-blinded randomized controlled evaluation of safety, immunogenicity and efficacy of MVA85A/AERAS-485 in BCG vaccinated infants without tuberculosis or HIV infection. Infants (126 to 182 days) will be randomized to receive a study vaccine (MVA85A/AERAS-485 or *Candida* skin test antigen control). The study is planned at a single site in South Africa but may be executed at more than one site. Enrollment is expected to be completed in 21 months. Given completion of enrollment in 21 months, all infants will be followed for at least 15 months after the last infant is enrolled into the study. The total duration of follow-up for each infant is at least 15 months and up to 39 months. Infants will be followed for the entire duration both for the development of tuberculosis and serious adverse events. A case of tuberculosis will be defined using a composite endpoint derived from epidemiological cohort studies in BCG-vaccinated infants.

Male or female infants entered into the study will receive a conjugated pneumococcal vaccine (Prevenar®) with the scheduled DPT immunizations Study Day -84 through Study Day -14. On enrollment to the study, eligible infants will be assigned to a study group starting with Study Group 1 and they will be randomly assigned within a study group to receive either MVA85A/AERAS-485 or *Candida* skin test antigen control as shown below. Infants will be assigned to a safety cohort (Study Group 1), then into one of three immunological assay evaluation groups (Study Groups 2-4), and finally the remainder of infants will be assigned into the correlate of protection cohort (Study Group 5). At least 330 infants will be randomized in Study Group 1, up to 50-60 infants each in Study Groups 2-4, and the remaining infants will be randomized in Study Group 5 such that the total number of infants randomized on the study will be 2784.

Study Group	Cohort Evaluation	MVA85A/ AERAS-485 (ID)	Control *Candida* skin test (ID)	N
1	Safety (labs and adverse events)	165	165	330
2	ELISPOT Immunogenicity	30	30	60
3	ICS Assay Immunogenicity	30	30	60
4	Whole Blood Immunogenicity	30	30	60
5	Correlate of Protection	1137	1137	2274
	Total	1392	1392	2784

The sample sizes were selected because they are judged to be adequate for safety evaluations. A sample size of 1392 MVA85A/AERAS-485 infants will give a greater than 75% chance of observing an adverse event that has an approximately 1 in a 1000 actual rate of occurrence. A sample size of 165 MVA85A/AERAS-485 infants in the specialized safety cohort will provide an 80% chance of observing at least one adverse event which has an approximate 1% actual rate of occurrence. Given a tuberculosis cumulative incidence of 3% over 18 months in the control group, 1392 infants per study group will provide a 90% chance of detecting a 60% reduction between the treated and control groups based on a two-sided log rank test at a significance level of 0.05.

2.2.1 Rationale for Blinding Procedures

This study will investigate both safety and efficacy endpoints in a vulnerable population: infants. In order to provide both MVA85A/AERAS-485 and control groups intended benefit, Prevenar has been specified as a study vaccine. Prevenar is a licensed vaccine and is required to be given to all those infants without contraindications who have completed a consent agreement for the study.

MVA85A/AERAS-485 is a difficult vaccine to blind because it is an intradermal vaccine and it causes a skin reaction as an expected characteristic of its administration. As a result of this difficulty a double-blinded evaluation of MVA85A has never been performed. Because of the imperative need to collect unbiased information both on safety and efficacy of this prospective tuberculosis vaccine, an intradermal control injection (0.06 mL, less than the volume of a tuberculin test) with a *Candida* skin test antigen has been included in this protocol. *Candida* is fungus that is a normal part of human flora. It can be found on all human bodies. Thus all humans typically have developed protective immunity to it. The *Candida* skin test reaction is similar to a tuberculin skin test in almost every way except it is directed at *Candida* antigens rather than tuberculosis antigens. This skin test product has been used widely to assess cellular immunity in HIV patients and although not sold as a pediatric product has been used safely in infants (see investigator's brochure) as have other skin test allergens. Despite a general avoidance of inactive controls in pediatric research subjects, the benefit of collecting unbiased information was deemed to be greater than any potential risk from this widely used antigen.

3 STUDY PROCEDURES

3.1 Summary Schedule of Subject Evaluations

Infants who are randomized at Study Day 0 will be followed up on a regular schedule at Study Day 7 (±2 days), Study Day 28 (± 5 days), Study Day 84 (± 5 days) and every 84 days (± 7 days) until the end of the study. Specific evaluations at each follow-up will be determined by the study group to which an infant is enrolled as described below. Parents of infants will also be requested to consent to have <1 mL of blood collected by heel prick at Study Day 1; consent will be sought for subjects in any of the five study groups until blood from 100 infants is collected. This additional blood collection is optional and a separate consent will be required. The blood will be used to evaluate the innate immune response post-vaccination by transcriptional analysis of whole blood.

Study Group 1 - Safety Cohort

At least 330 infants will be assigned to the Safety cohort (Study Group 1). Infants in Study Group 1 will be followed according to Table 1. When the last infant in this cohort completes Study Day 84, the Safety Monitoring Committee will evaluate the unblinded safety data to determine if a pattern of adverse events related to MVA85A/AERAS-485 or other safety concerns exists. If such a pattern exists, the Safety Monitoring Committee will determine if additional infants will be entered into this safety group for additional safety determination or if further studies need to be performed.

Table 1 Infant Schedule of Evaluations and Treatments Study Day -84 to Study Day 84 for the Safety Cohort

Study Day	-84[a]	-35[a]	-14[a]	0	7	28	84
Minimum Age (Days)	42	91	112	126	133	154	210
Written Informed Consent	X						
Verify Eligibility Criteria				X			
Medical History		X					
Physical Examination		X					
HIV-1			3				
Vital Signs				X	X	X	X
Weight		X		X	X	X	X
Injection Site Photograph				X			
Study Vaccine Administration				X			
Serum Chemistry[b]			1.0		1.0	1.0	
CBC, Differential, Platelets			1.0		1.0	1.0	
QuantiFERON (Gold in tube)			3				
Immunology Samples						8	
Solicited Adverse events				X	X		
Assess Adverse Events				X	X	X	
Serious Adverse Event Reports				X	X	X	X
Site of Injection Examination				X	X	X	
Blood volume (mL) per visit			8.0		2.0	10.0	

[a] Written informed consent must be obtained prior to initiation of study screening activities and may be obtained prior to Day -84. Medical history and physical exams must be completed within 35 days prior to Study Day 0 and baseline safety blood collections must be completed within 14 days prior to randomization. If the serum chemistry and/or CBC, differential, and/or platelets are repeated prior to randomization, the QuantiFERON test does not need to be repeated.

[b] GGT, ALT, AST, total bilirubin, creatinine, Alkaline phosphatase

Study Group 2- ELISPOT Immunogenicity Cohort

The next 50-60 infants enrolled will be assigned to the ELISPOT Immunogenicity Cohort. Table 2 contains the schedule of evaluations for this cohort.

Table 2 Infant Schedule of Evaluations and Treatments Study Day -84 to Study Day 84 for ELISPOT Immunogenicity Cohort

Study Day	-84[a]	-35[a]	-7[a]	0	7	28	84
**Minimum Age (Days)**	**42**	**91**	**119**	**126**	**133**	**154**	**210**
Written Informed Consent	X						
Verify Eligibility Criteria				X			
Medical History		X					
Physical Examination		X					
HIV-1		3					
Vital Signs				X	X	X	X
Weight		X		X	X	X	X
Injection Site Photograph				X			
Study Vaccine Administration				X			
QuantiFERON (Gold in tube)		3					
Immunology Samples			5		5		
Solicited Adverse events				X	X		
Assess Adverse Events				X	X	X	
Serious Adverse Event Reports				X	X	X	X
Site of Injection Examination				X	X	X	
Blood volume (mL) per visit		6	5		5		

[a] Written informed consent must be obtained prior to initiation of study screening activities and may be obtained prior to Day -84. Medical history/physical exams and blood collection for HIV-1 and QuantiFERON testing must be completed within 35 days prior to Study Day 0; blood collections for baseline immunology must be completed within 7 days prior to randomization.

Study Group 3 – ICS Assay Immunogenicity Cohort

The next 50-60 infants enrolled will be assigned to the ICS Assay Immunogenicity Cohort. Table 3 contains the schedule of evaluations for this cohort.

Table 3 Infant Schedule of Evaluations and Treatments Study Day -84 to Study Day 84 for the ICS immunogenicity Cohort

Study Day	-84[a]	-35[a]	-7[a]	0	7	28	84
Minimum Age (Days)	42	91	119	126	133	154	210
Written Informed Consent	X						
Verify Eligibility Criteria				X			
Medical History		X					
Physical Examination		X					
HIV-1		3					
Vital Signs				X	X	X	X
Weight		X		X	X	X	X
Injection Site Photograph				X			
Study Vaccine Administration				X			
QuantiFERON (Gold in tube)		3					
Immunology Samples			8			8	
Solicited Adverse events				X	X		
Assess Adverse Events				X	X	X	
Serious Adverse Event Reports				X	X	X	X
Site of Injection Examination				X	X	X	
Blood volume (mL) per visit		6	8			8	

[a] Written informed consent must be obtained prior to initiation of study screening activities and may be obtained prior to Day -84. Medical history/physical exams and blood collection for HIV-1 and QuantiFERON testing must be completed within 35 days prior to Study Day 0; blood collections for baseline immunology must be completed within 7 days prior to randomization.

<u>Study Group 4 – Whole Blood Assay Cohort</u>

The next 50-60 infants enrolled will be assigned to the Whole Blood Immunogenicity Assay Cohort. Table 4 contains the schedule of evaluations for this cohort.

Table 4 Infant Schedule of Evaluations and Treatments Study Day -84 to Study Day 84 for the Whole Blood Immunogenicity Assay Cohort

Study Day	-84[a]	-35[a]	-7[a]	0	7	28	84
Minimum Age (Days)	42	91	119	126	133	154	210
Written Informed Consent	X						
Verify Eligibility Criteria				X			
Medical History		X					
Physical Examination		X					
HIV-1		3					
Vital Signs				X	X	X	X
Weight		X		X	X	X	X
Injection Site Photograph				X			
Study Vaccine Administration				X			
QuantiFERON (Gold in tube)		3					
Immunology Samples			6			6	
Solicited Adverse events				X	X		
Assess Adverse Events				X	X	X	
Serious Adverse Event Reports				X	X	X	X
Site of Injection Examination				X	X	X	
Blood volume (mL) per visit		6	6			6	

[a] Written informed consent must be obtained prior to initiation of study screening activities and may be obtained prior to Day -84. Medical history/physical exams and blood collection for HIV-1 and QuantiFERON testing must be completed within 35 days prior to Study Day 0; blood collections for baseline immunology must be completed within 7 days prior to randomization.

Study Group 5 - Correlate of Protection cohort

After Study Groups 1-4 have completed enrollment, infants will be enrolled into Study Group 5 until the total sample size of 2784 infants is reached. Table 5 contains the schedule of evaluations for this cohort.

Table 5 Infant Schedule of Evaluations and Treatments Study Day -84 to Study Day 84 for the Correlate of Protection Cohort

Study Day	-84[a]	-35[a]	-7[a]	0	7	28	84
Minimum Age (Days)	42	91	119	126	133	154	210
Written Informed Consent	X						
Verify Eligibility Criteria				X			
Medical History		X					
Physical Examination		X					
HIV-1		3					
Vital Signs				X	X	X	X
Weight		X		X	X	X	X
Injection Site Photograph				X			
Study Vaccine Administration				X			
QuantiFERON (Gold in tube)		3					
Immunology Samples			8			8	
Solicited Adverse events				X	X		
Assess Adverse Events				X	X	X	
Serious Adverse Event Reports				X	X	X	X
Site of Injection Examination				X	X	X	
Blood volume (mL) per visit		6	8			8	

[a] Written informed consent must be obtained prior to initiation of study screening activities and may be obtained prior to Day -84. Medical history/physical exams and blood collection for HIV-1 and QuantiFERON testing must be completed within 35 days prior to Study Day 0; blood collections for baseline immunology must be completed within 7 days prior to randomization.

After study day 84 study enrollees will be visited by a study member at least every 84 days until the end of the study. At these visits, infants will be weighed, assessed for the occurrence of a new SAE, and assessed for admission to a case verification ward for suspected tuberculosis. Any infant with a cohabiting household member in which the diagnosis of tuberculosis has been made or any infant that has 1) weight loss in last two months, 2) cough for more than two weeks without improvement, 3) failure-to-thrive, or 4) conversion to a positive QuantiFERON test or positive Mantoux test will be admitted to the case verification ward for diagnosis of tuberculosis.

A QuantiFERON test will be drawn on Study Day 336 for all subjects followed to Study Day 336 and on the last clinic visit in all infants without the clinical diagnosis of tuberculosis. Follow up of all infants will be terminated at the next scheduled study visit after the last enrollee completes 15 months of follow-up (Study Day 420).

3.2 Subject Selection

3.2.1 Recruitment and Informed Consent

Parents/guardians bringing their infants for routine immunizations or other visits at public sector clinics will be asked if they are interested in entering their infant in this research

protocol. Study staff may also approach parent/guardians of infants at home using information obtained from clinic records, birth records, word of mouth referrals from community contacts, or after direct contact parents/guardians. They will be informed about the study including the inclusion/exclusion criteria of the study. Written informed consent will be obtained prior to conducting any study-related procedures using a consent form approved by the IRB and signed and dated by the parent/guardian at the time of consent. The clinical investigator, or designee, will conduct the consent discussion on an individual basis with each parent/guardian and will allow adequate time for all questions to be addressed. A copy of the signed consent form will be given to the parent/guardian; the parents/guardians' willingness to continue on the study will also be assessed at each study-related screening visit.

3.2.2 Screening

Infants whose parents/guardian have signed a consent will be screened to assess eligibility for the study. A screening log will be maintained by the site that records all infants whose parents/guardian signed an informed consent and entered the screening process. The screening log will document the date of consent, initials, infant's date of birth, race/ethnicity and gender. For screened individuals who are subsequently randomized in the study, the date of randomization and the assigned subject identification number will also be recorded. For screened individuals who are not subsequently randomized in the study, the screening log will also provide details as to why an individual did not participate in the study. Abnormal results and findings that make the infant ineligible will be discussed with the infant's parent/guardian and the infant will be referred for follow-up care with their healthcare provider if necessary.

3.2.3 Subject Eligibility (Inclusion/Exclusion Criteria)

Eligibility for randomization into the study at Study Day 0 will be based on the inclusion and exclusion criteria described below. The investigator must document confirmation of eligibility immediately prior to randomization.

3.2.3.1 Inclusion Criteria

Male and female infants are eligible for the study and must meet **all** of the following criteria:

1. Age of 126 through 182 days on the day of randomization (Study Day 0)
2. Written informed consent obtained from the parents/guardian
3. Weight: by chart >3rd percentile on Study Day 0 or, if < 3rd percentile, infant has shown a stable growth pattern
4. BCG vaccination within 7 days of birth.
5. Generally good health confirmed by medical history and physical examination within 35 days prior to Study Day 0
6. Must have received age-appropriate doses of pneumococcal vaccine as recommended by the South African Department of Health but no injection within 14 day prior to Study Day 0
7. Ability to complete follow-up period as required by the protocol
8. Completed simultaneous enrollment in the Aeras Vaccine Development Registry protocol

3.2.3.2 Exclusion Criteria

Infants must **not** have any of the following:

1. Acute illness on Study Day 0
2. Fever ≥37.5°C on Study Day 0
3. Evidence of significant active infection on Study Day 0
4. Received a EPI immunization within 14 days prior to Study Day 0
5. Historical or virological evidence of individual or maternal human immunodeficiency virus (HIV-1) infection
6. History of allergic disease or reactions likely to be exacerbated by any component of the study vaccine
7. Previous medical history, or evidence, of an intercurrent illness that may compromise the safety of the infant in the study
8. Evidence of chronic hepatitis from any cause
9. History or evidence of any systemic disease on physical examination or any acute, chronic or intercurrent illness that, in the opinion of the investigator, may interfere with the evaluation of the safety or immunogenicity of the vaccine
10. History of or known tuberculosis or treatment for tuberculosis
11. Shared residence since birth with an individual with active tuberculosis or on anti-tuberculosis treatment for less than 2 months

3.2.3.3 Screening Clinical Assessments and Laboratory Tests

All infants whose parents/guardians have signed an informed consent will begin a regimen of vitamin and iron therapy approximately 35 days prior to Study Day 0 to insure that they are metabolically and hematopoietically competent to participate in the trial. All infants will receive a conjugated pneumococcal vaccine with the scheduled DPT immunizations Study Day -84 through Study Day -14. Infants who have not received conjugated pneumococcal vaccine injections at least 14 day prior to prior to Study Day 0 will not be eligible for randomization. Prevenar and the rotavirus vaccine, Rotarix®, will be made available at all health clinics for all infants in the study area regardless of study enrollment until the vaccines become routinely available from the local health departments.

Infants will be medically evaluated within 35 days prior to receipt of study vaccine/control to ensure that their state of health will not expose them to increased risk from protocol participation compared to other infants in good health.

3.3 Assignment to Study Group and Randomization

Infants will be randomized in a 1:1 ratio to MVA85A/AERAS-485 or control within each Study Group using an interactive voice/web response system (IVRS/IWRS). The randomization schedule will be prepared by a statistician who will not be involved with the analysis of the study in order to maintain blinding of the study team.

3.3.1.1 Study Blind

The MVA85A/AERAS-485 and the *Candida* skin test antigen control are packaged and labeled to appear indistinguishable from each other at the time of administration.

All source documentation and dose preparation records must remain in a secure location with access limited to the study vaccine manager and the study monitor for the entire study period or until the clinical site is notified by Aeras or its designee that the study has been unblinded.

3.3.1.2 Requests for Unblinding

If there is an urgent clinical requirement to know an infant's treatment assignment, the investigator (in consultation with the medical monitor, if possible) will make a written request to the vaccine manager or designee. The request must include the subject identification number, the date, a brief justification of the clinical requirement to the vaccine manager in the research pharmacy, and the investigator's signature. The request will be kept in the study file.

Upon receipt of proper written request, the vaccine manager or designee will disclose the treatment assignment to the investigator. Aeras must be notified immediately of any clinically required break of the study blind on an Immediately Reportable Event Form.

3.4 Study Vaccination

Infants must receive their dose of study vaccine/control on Study Day 0 as soon as possible after randomization. Before administering any injections, the study team member in the clinic who will be administering the vaccine/control must inspect the syringe and volume, checking that the syringe is identified with the correct subject identification number and initials and checking the date and time the dose was prepared. If circumstances result in a delay of administration beyond the allotted time, an explanation must be entered into the source documents and the expired syringe must be returned to the study vaccine manager, who will prepare a replacement syringe. The study vaccine manager will dispose of the expired syringe only upon authorization from the study monitor. If administration of study vaccine/control does not occur on the day of randomization, the principal investigator must notify the sponsor immediately.

The vaccine/control will be administered intradermally into the skin over the deltoid region of the opposite arm to which BCG was previously administered. The investigator will wear gloves and eye protection. Infants will stay in the unit for 60 minutes after vaccination for observation. As with any vaccine, allergic reactions to dose administration are possible. Therefore, appropriate drugs and medical equipment to treat acute anaphylactic reactions must be immediately available and a medically qualified study team member trained to recognize and treat anaphylaxis must be present in the clinic during the entire vaccination procedure and post-vaccination monitoring period.

In order to minimize dissemination of the recombinant vectored vaccine virus into the environment the inoculation site will be covered with a dressing after immunization. This should absorb any virus that may leak out through the needle track. The dressing will be removed from the injection site at the end of the 60 minute observation period and will be

disposed as GMO waste by autoclaving, in accordance with the relevant SOP and current local practice.

Administration of the study vaccine/control (including date, time, and which arm was injected) must be documented in the infant's study records by the study team member who administered the vaccine/control.

3.5 On-study Visit Procedures

All infants who are randomized and receive study vaccine/control will continue to be followed according to the protocol visit schedule of the study group to which they are assigned, unless consent is withdrawn. All infants randomized and who receive study vaccine will, however, continue to be followed on the Aeras Vaccine Development Registry protocol after completing C-020-485.

All deviations from protocol procedures, evaluations, and/or visits must be documented as they occur. Missed evaluations must be rescheduled or performed at the nearest possible time point to the original schedule.

3.5.1 Pre-vaccination and Post-vaccination Monitoring of Subjects

Infants will remain under close observation for at least 60 minutes after receiving study vaccine/control. Infants will have vital signs (pulse and axillary temperature) taken prior to vaccine/control administration; vital signs will be repeated before infants leave the clinic.

3.5.2 Clinical Assessments and Laboratory Tests

An examination of the injection site and draining lymph nodes of injection arm will be performed 60 minutes after study vaccine/control administration and at Study Days 7 and 28. Infants will have vital signs recorded at Study Days 7, 28, and 84. Infants will also have their weight recorded prior to vaccination on Study Day 0 and then again on Study Day 7, 28, 84 and every 84 days thereafter.

Laboratory evaluations are planned for only the infants enrolled in the Safety Cohort (Study Group 1). The Safety Monitoring Committee may determine that additional infants need to enroll in this cohort after a review of the safety data from these infants. Blood for hematology and serum chemistry will be collected at screening and on Study Days 7 and 28. All laboratory samples will be processed according to SOPs provided by the local clinical laboratory.

Prior to any blood draw, all infants will be evaluated for clinical signs of anemia (e.g., palmar pallor). If the evaluation suggests anemia in an infant, the infant will have hemoglobin evaluated by fingerprick. If the hemoglobin obtained by fingerprick is < 9g/dL, then blood will not be drawn from that infant at that visit.

All infants randomized to the study will have blood for QFT-G collected during screening, again on Study Day 336, during evaluations for TB in the CV ward, and on the last scheduled visit for each infant.

3.5.2.1 Abnormal Clinical Laboratory Test Results

Results from clinical laboratory tests obtained on the study must be reviewed by the investigator (or a designee who is a medically qualified study team member) within 72 hours of receiving the results to determine if abnormalities exist. If the laboratory value is abnormal, it must be reported as an adverse event and repeated promptly to demonstrate resolution. Additional laboratory tests may be performed if the investigator deems them to be necessary to fully evaluate an adverse event. In the event that the investigator elects to order non-protocol-specified laboratory tests, the investigator must record the rationale for the tests and a determination of clinical significance of the result in the source documents. The investigator must keep the medical monitor informed of adverse events of clinical significance.

Abnormal results and findings will be discussed with the infant's parents/guardians, or the infant will be referred for follow-up with their healthcare provider if necessary.

3.5.3 Immunology Laboratory Evaluations

Staff at the clinical research site will refer to the most current version of the Specimen Management Manual (provided under separate cover) for instructions and additional information on specimen collection and processing. Processing of blood samples for immunologic evaluations for each infant will depend on the study group to which the infant is enrolled. Cells from blood samples collected from infants in Safety Cohort (Study Group 1) and the Correlation of Protection Cohort (Study Group 5) will be stored frozen and tested after the end of the study. Plasma and cells remaining from the immunologic assays in Study Groups 1-5 will be stored frozen for possible future testing to evaluate immune responses.

The amount of blood required for this study is within guidelines for blood donation. See Appendix B, Phlebotomy Analysis, for blood volumes collected at each study visit and in total for the study.

3.5.4 Safety Evaluations

Through the 28-day period following vaccination, all **adverse events** observed by the investigator (or designee) or reported by the infant's parents/guardians spontaneously in response to a direct question or recorded on the diary card, will be medically evaluated and documented in terms of a medical diagnosis on the Adverse Event CRF.

Through the entire study period, all **serious adverse events** observed by the investigator or reported by the infant's parents/guardians spontaneously or in response to a question will be medically evaluated and documented on the appropriate adverse event CRF.

3.5.5 Concomitant Medications

The collection of information on concomitant medications used by infants following vaccination will coincide with the collection period of adverse events. The collection period for concomitant medications associated with treatment of adverse events will be 28 days following vaccination. The collection period of concomitant medications associated with the treatment of serious adverse events (SAE) will be from Study Day 0 through the end of the study.

Concomitant medication includes prescription and non-prescription drugs or other treatments, and any vaccines other than the study vaccine. The names of medication, treatment start and stop dates (or 'ongoing'), route of administration, and indication must be recorded on the Concomitant Medications case report form (CRF). The indication recorded on the Concomitant Medications CRF must correspond to a medical term/diagnosis recorded on the adverse event (AE) CRF unless the indication is not an AE or an SAE, or to a pre-existing condition noted in the infant's medical history, or as prophylaxis, e.g., dietary supplement.

3.5.6 Diagnosis of Tuberculosis and Case Verification

Any infant with a cohabiting household member in which the diagnosis of tuberculosis has been made or any infant that has 1) weight loss in last two months, 2) cough for more than two weeks without improvement, 3) failure-to-thrive, or 4) conversion to a positive QuantiFERON test or positive Mantoux test will be admitted to the case verification ward for diagnosis of tuberculosis.

Asymptomatic children will be admitted to the case verification ward for investigation once for each new cohabiting household tuberculosis contact identified and will be readmitted only if they have continued exposure to an inadequately treated cohabiting household tuberculosis contact. Symptomatic children will be readmitted to the case verification ward if they have new symptoms, or if they were not previously treated for tuberculosis disease and no alternative diagnosis of their symptoms was made, or if a previous course of anti-tuberculous treatment has since been completed. Children who are receiving prophylactic isoniazid monotherapy may be readmitted to the case verification ward for investigation.

On admission, a relevant medical history for differential diagnosis of TB and a physical examination will be performed and a tuberculin skin test will be administered by the Mantoux technique. The next morning, an early morning gastric washing will be collected for TB smear and culture and an induced sputum specimen will be obtained for TB smear and culture. This will be repeated the next morning and the results of the tuberculin skin test will be read prior to discharge that morning. A chest-radiograph will be obtained on admission. Blood will be drawn for HIV testing and other blood tests that are clinically indicated (e.g., CBC), as well as for tuberculosis immune assays (i.e., QuantiFERON).

All positive cultures will be tested with PCR for speciation to exclude MOTT and BCG disease. Drug sensitivities of positive cultures will also be obtained. Some of the cultures may, in addition, undergo molecular testing to identify which substrain of organism is present.

The results of all examinations will be summarized and provided to the parent/guardian in a letter. If a diagnosis of TB is made or an infant is in close contact with an adult diagnosed with TB (including infants identified during screening), a letter will also be sent to the TB medical officer in the area where the child lives with all test results and a recommendation on treatment. If a diagnosis of TB is made or an infant is in close contact with an adult diagnosed with TB (including infants identified during screening), the parent/guardian will be given a one-week supply of anti-TB medication and instructed to contact their local clinic, which will be responsible for ongoing management according to current South African national TB programme guidelines.

The hospital records of any children enrolled in the study who may be diagnosed with TB disease as part of routine care in other health facilities will be reviewed to determine if the clinical diagnosis meets the study case definition of TB disease, as defined by the TB Case Definition Endpoints in Section 7.3.1.

3.5.7 Subject Follow-up and Contact

Parents/guardians of infants will be instructed to contact a study team member to report new diagnoses or new or worsening adverse events and to come to the study clinic if medical attention is needed, provided the urgency of the situation permits. For emergencies and other unscheduled visits to a medical facility other than the study clinic, medical records will, to the extent possible, be obtained by the investigator.

During each visit, parents/guardians of infants will be reminded to notify a study team member of the following:

- The occurrence of adverse events through the 28 days following vaccination and the occurrence of serious adverse events through the end of each infant's follow-up
- Receipt of any concomitant medications
- Plans to move or if contact information changes
- If infant's parents/guardian have decided to withdraw from the study
- Change in general health status
- Any other change in status that may affect the infant's participation (e.g., plan to participate in another investigational study)

3.5.8 Subject Participation and Loss to Follow-up

If the site's study team members are unable to establish contact with an infant who misses a scheduled study visit, the clinical site must make every possible effort to contact the infants and document such efforts. If contact is re-established, then the infant will resume participation.

If contact with the infant cannot be re-established by the infant's calculated last study visit, then a determination of "lost to follow-up" can be made.

4 STUDY VACCINES

Staff at the clinical research site will refer to the most current version of the Study Vaccine Management Manual (provided under separate cover) for further instructions on vaccine/control storage and preparation. Additional information is also provided in the Investigator's Brochure.

4.1 Supplies

Aeras will provide the study vaccine manager with adequate quantities of MVA85A/AERAS-485 and control for the study prior to the start of vaccination.

MVA85A/AERAS-485 is manufactured in accordance with GMP by Impfstoffwerk Dessau-Tornau (IDT) Biologika GmBH, Am Pharmapark, D-06861 Dessau-Rosslau, Germany. A description of the contents of the vials follows.

MVA85A/AERAS-485: formulated in Tris buffer (10 mM Tris, 140 mM NaCl, pH 7.7) at a concentration of 1.89 x 10^9 plaque-forming units (pfu)/mL. The dose of study vaccine to be administered will be approximately 1 x10^8 pfu (0.06 mL injected intradermally).

Descriptions of Prevenar (manufactured by Wyeth Pharmaceutical Inc. Philadelphia, PA, USA) and the *Candida* skin test antigen control (manufactured by Allermed Laboratories, Inc., San Diego, CA, USA) can be found in the MVA85A/AERAS-485 Investigator's Brochure.

4.2 Accountability

The study vaccine manager is required to maintain accurate study vaccine (i.e., MVA85A/AERAS-485 and Candin) accountability records. Instructions and required forms to be completed and kept for accountability will be provided to the study vaccine manager. Upon completion of the study, all original study vaccine management records will be returned to Aeras or its designee. Copies of the study vaccine management records must be maintained with the rest of the study records.

4.3 Receipt and Storage

Upon receipt of study vaccine supplies, the study vaccine manager must immediately inspect all vials for damage. Study vaccine/control will be shipped with a continuous temperature-monitoring device. The temperature of the shipment must be documented by the study vaccine manager. Any deviations or problems identified must be documented and promptly discussed with Aeras and the study monitor to determine the usability of the supplies or if replacements must be sent.

Study vaccine/control must be stored in a secured location with no access for unauthorized personnel. Study vaccine/control should be placed in a container clearly marked "For Aeras C-020-485 Study Only".

Study vaccine will be stored frozen at -20° C or colder in a frost-free or frostless freezer; the *Candida* antigen control will be stored at 2-8º C. Storage temperature must be monitored

continuously and a log of the monitored temperature maintained. The freezer must be equipped with a continuous temperature monitor (wheel device) or should be alarmed in case the temperature exceeds specified ranges. Complete storage instructions will be provided in the Study Vaccine Management Manual.

4.4 Vaccine Preparation and Dose

Preparation of the study vaccine/control for injections should not begin before an infant is confirmed eligible on Study Day 0. Infants must also be in the clinic on these days and must agree to receive the vaccine/control on the same day.

The study vaccine manager will follow the detailed instructions provided in the Study Vaccine Management Manual to prepare the doses of study vaccine/control using aseptic technique. One vaccine vial per infant to be immunized will be used; a vial of *Candida* control antigen may be used to prepare injections for more than one infant. The study vaccine will be allowed to thaw to room temperature before being withdrawn into a masked syringe and administered within 2 hours. The study vaccine manager will draw 0.06 mL of study vaccine/control into the masked syringe, will label the syringe with the infant's identification number and initials, the date and time of preparation, and the volume in the syringe.

Precautions should be taken to avoid contact of the study vaccine with broken skin, and gloves and eye protection (safety goggles) should be worn.

Preparation of the vaccine/control including date and time must be documented in the study vaccine management records by the study vaccine manager who prepared the doses.

4.5 Disposition of Unused Supplies

All unused study vaccine and supplies will be returned to Aeras at the end of the study or disposed of upon authorization from Aeras or according to the facility's SOPs. Any disposing of study vaccine conducted at the clinical site will be documented in the study file.

5 SAFETY

5.1 Responsibilities for Insuring the Safety of Trial Subjects

The national regulatory authority, the sponsor (Aeras), the institution through which the research is performed and all members of the principal investigator's clinical team share responsibility for insuring that participants in this trial are exposed to the least possible risk of adverse events that may result from participation in this protocol.

5.1.1 Principal Investigator

The principal investigator has a personal responsibility to closely monitor trial subjects and an inherent authority to take whatever measures necessary to insure their safety. The principal investigator has the authority to terminate, suspend or require changes to a clinical trial for safety concerns and may delay an individual's study vaccine administration or pause study

vaccine administration in the whole trial if the investigator has some suspicion that the study vaccine might place a subject at significant risk. The principal investigator determines severity and causality with respect to the study vaccine for each adverse event. For blinded studies the principal investigator is blinded, in which case the study vaccine may consist of either a placebo, an active control, or the investigational product.

5.1.1.1 Study Vaccine Pausing Rules for the Principal Investigator

If the principal investigator determines that a suspected unexpected serious adverse reaction (SUSAR) OR a serious adverse event OR an adverse event pattern of concern that is judged to be POSSIBLY, PROBABLY or DEFINITELY related to study vaccine has occurred, the principal investigator will pause administration of study vaccine in the trial. If the principal investigator pauses study vaccine administration he or she will record this in a memorandum to the study file and notify the sponsor.

If the principal investigator pauses study vaccine administration in a trial under the rules in this section and additional clinical information becomes available that reduces the principal investigator's assessment of causality, severity or toxicity grade such that the adverse event's causality, severity or toxicity grade no longer requires pausing then the principal investigator, with the agreement of the local medical monitor, may resume study vaccine administration with a memorandum to the study file and notification of the sponsor.

5.1.2 Study Sponsor

The sponsor (Aeras) also has an institutional responsibility to insure subject safety. This responsibility is vested in two medical monitors (one local medical monitor and one global medical monitor) and a safety monitoring committee (SMC).

5.1.3 Local Medical Monitor

The local medical monitor is the sponsor's representative and is a licensed physician or surgeon in the country of residence. The local medical monitor reviews the safety of the product for protocols in a specific region and determines expectedness of the adverse event. The local medical monitor may make a sponsor's assessment of severity and causality for adverse events that may upgrade the degree of severity and causality determined by the principal investigator. The local medical monitor, like the principal investigator, is blinded for a blinded study.

5.1.3.1 Study Vaccine Pausing Rules for the Local Medical Monitor

If the local medical monitor determines that a SUSAR OR a serious adverse event OR an adverse event pattern of concern that is judged to be POSSIBLY, PROBABLY or DEFINITELY related to study vaccine has occurred, the local medical monitor will pause administration of study vaccine in the trial. If the local medical monitor independently pauses administration of study vaccine in the study, he or she will record this in a memorandum to the study file and notify the principal investigator and sponsor who will then convene the SMC. In all cases administration of study vaccine may resume only if permitted by the SMC.

Any changes to the protocol required by the SMC as a condition of study vaccine resumption must be approved by, or submitted to, the institutional review board/ethical committee and the national regulatory authority.

5.1.4 Global Medical Monitor

A global medical monitor is retained by Aeras through the contract research organization PPD, Inc., located in Cambridge, UK. The global medical monitor reviews all SUSAR reports and since the global medical monitor is unblinded s/he determines if a report is expedited since only reports associated with an investigational agent (not placebo) are expedited. The global medical monitor may determine that the subject received placebo thus the report is entered into the database with the suspect product placebo but is not expedited and does not need to be forwarded as an expedited report. The reporting situation with active controls is different since events associated with active controls may need to be reported under post-marketing pharmacovigilance regulations.

5.1.4.1 Study Vaccine Pausing Rules for the Global Medical Monitor

Since the global medical monitor is unblinded and reviews data from all Aeras trials, he or she may become aware of an adverse event pattern of concern not appreciated by the principal investigator or local medical monitor. If the global medical monitor independently determines that an adverse event pattern of concern that is judged to be POSSIBLY, PROBABLY or DEFINITELY related to study vaccine has occurred, the global medical monitor will pause administration of study vaccine in the trial. If the global medical monitor pauses administration of study vaccine in the trial he or she will record this in a memorandum to the study file and notify the local medical monitor, principal investigator and sponsor who will convene the SMC. In all cases administration of study vaccine may resume only if permitted by the SMC. Any changes to the protocol required by the SMC as a condition of study vaccine resumption must be approved by, or submitted to, the institutional review board/ethical committee and the national regulatory authority.

5.1.5 Safety Monitoring Committee

If study vaccine administration is paused by the principal investigator, the local medical monitor, or the global medical monitor, an SMC will be convened. The SMC is composed of five voting members (two licensed physicians from South Africa, one licensed physician from the United Kingdom, and two licensed physicians from the US) and may have up to two alternate physician members. The voting members can not be directly involved with the conduct of the study, can not be employees of Aeras and must be experienced in the evaluation of adverse events in clinical trials. The SMC will also have a non-voting statistician and a non-voting Aeras member who will record committee actions.

The SMC will convene to review safety data when the last subject in the Safety Cohort (Study Group 1) completes Study Day 84. The SMC will evaluate blinded and unblinded-by-group safety data to determine if a pattern of adverse events related to MVA85A/AERAS-485 or other safety or risk concerns exist. Only the SMC and the independent statistician responsible for preparing these analyses would be unblinded during these reviews. If such a pattern

exists, the Safety Monitoring Committee will determine if additional subjects will be entered into this safety group for additional safety determination or if further studies need to be performed. The SMC will conduct a second unblinded-by-group safety and risk review after the 1000th infant completes the Study Day 84 visit. The SMC may also review an individual SAE or it may choose to review adverse events, serious adverse events, solicited adverse events, and laboratory and vital signs data. The SMC may unblind any amount of safety information needed to conduct their assessment. All procedures associated with this review, including objectives, data handling, and elements to be included for review will be documented in SMC minutes.

Based on its review and the protocol stopping rules (Section 6) the SMC will make recommendations in the SMC minutes to Aeras regarding further conduct of the study and further administration of study vaccine. The conclusions of the SMC will be communicated to the investigators and the IRB/Ethical Committees and the national regulatory authority for their information. The sponsor agrees to abide by the decision of its SMC.

5.1.6 Institutional Review Boards and Ethics Committees

The Institutional Review Board or Ethics Committee has institutional responsibility for the safety of research subjects. The Institutional Review Board or Ethics Committee has the authority to terminate, suspend or require changes to a clinical trial.

5.1.7 National Regulatory Authority

Since the national regulatory authority (such as the FDA for the U.S., or the MCC of South Africa) receives all expedited reports it also has the authority to terminate, suspend or require changes to a clinical trial.

5.2 Safety Surveillance During the Study

Subjects will be monitored and safety data collected by way of clinical interviews with parents/guardians, examinations, and evaluations of daily diaries conducted by study team members and through reports of laboratory evaluations.

Through the 28-day period following each vaccination all **adverse events** observed by the investigator (or designee) or reported by the infant's parents/guardians spontaneously in response to a direct question or recorded on the diary card, will be medically evaluated and documented in terms of a medical diagnosis on the Adverse Event CRF.

Through the entire study period all **serious adverse events** observed by the investigator (or designee) or reported by the infant's parents/guardians spontaneously or in response to a question will be medically evaluated and documented on the appropriate adverse event CRF.

5.3 Definition of Adverse Event

An adverse event (AE) is defined as any unanticipated problem involving risks to study participants or others. An adverse event, therefore, can be any unfavorable or unintended sign, symptom, disease, syndrome, abnormal laboratory finding, or concurrent illness that

emerges or worsens relative to the infant's pretreatment baseline, whether or not it is considered to be related to the medicinal product.

All conditions that exist prior to administration of the study vaccine (pre-existing conditions) will be recorded in the infant's medical history to establish baseline. Day-to-day fluctuations in pre-existing conditions that do not represent a clinically significant change in the infant's status will not necessarily be reported as adverse events.

Any adverse change from the infant's baseline condition (determined from screening evaluations conducted to confirm study eligibility) that occurs following the administration of the study vaccine will be considered an adverse event. This includes the occurrence of a new adverse event or the worsening of a baseline condition, whether or not considered related to the study vaccine. Intermittent conditions such as headaches may be present on Study Day 0 but may represent an adverse event if the intensity or duration of the event is worse than usual following receipt of study vaccine. Adverse events include but are not limited to: adverse changes from baseline that represent increases in toxicity grade according to the Toxicity Table (see protocol appendices), adverse changes in the general condition of the infant, signs and symptoms noted by the infant's caregiver, concomitant disease with onset or increased severity after study vaccine administration, and changes in laboratory safety parameters occurring after study vaccine administration.

The reporting period for all adverse events is specified in Section 3. Adverse events will be reported on the Adverse Event Case Report Form (CRF) using a recognized medical term or diagnosis that accurately reflects the event. Adverse event evaluations will be reviewed by the principal investigator or by a designated medically qualified practitioner. Adverse event CRF pages are to be completed by members of the study team designated in writing by the principal investigator. The onset and resolution dates of the event and action taken in response to the event will be documented. All adverse events must be followed until resolution is demonstrated or until the end of the appropriate AE reporting period. The resolution date will be recorded on the CRF as the last date on which the infant experienced the adverse event or, if unresolved by the last day of the appropriate reporting period, the resolution date will be recorded on the CRF as "ongoing." Information recorded on the CRF must be substantiated in the source documents. If an adverse event evolves into a condition that becomes "serious," it will be designated as serious on the Adverse Event CRF and a SAE Report (SAER) form will be completed.

5.4 Assessing Severity

The safety concepts of "severity" and "seriousness" are distinct concepts (see Section 5.8). Severity refers to a degree of clinical manifestation. "Seriousness" refers to defined outcomes from an adverse event. A severe adverse event is not always serious and a serious adverse event is not always severe.

For all adverse events, the investigator (or designee, who is a healthcare professional; is someone the investigator deems qualified to review adverse event information, to provide a medical evaluation of the event, and to classify the event based upon medical judgment and

the severity categories described below) is responsible for assessing the severity of the event and the causal relationship of the event to the study vaccine.

The **severity** of all adverse events, including clinical findings and abnormal laboratory values, will be classified as one of the following grades:

1. **Mild**
2. **Moderate**
3. **Severe**

A Toxicity Table is provided in the protocol appendices for the assessment of severity of specified adverse events. The Toxicity Table Adverse Event Grades do not correlate directly with the classical severity grades of mild, moderate and severe. FOR THE PURPOSES OF RECORDING EVENTS ON THE CRF, Toxicity Table Grade 1 events will be considered mild in severity, Toxicity Table Grade 2 events will be considered moderate in severity, and both Toxicity Table Grade 3 and 4 events will be considered as severe. In the Toxicity Table certain local reactions such as erythema (redness) and swelling are graded according to size. Laboratory values are graded according to level of deviation from the normal range.

For adverse events not listed in the Toxicity Table determination of severity requires some level of interpretation as outlined below. The degree of incapacity caused by the adverse event and the level of medical intervention required for treatment may be helpful in assessing the overall severity of the adverse event.
For example:

- "Mild" events are generally regarded as noticeable but have no impact on normal activities; they may or may not require over-the-counter treatment managed by the infant.
- "Moderate" events generally have some impact on an individual's normal activities and may require general symptomatic medical intervention by a healthcare professional or by the infant.
- "Severe" adverse events may be incapacitating, leading to suspension of normal daily activities, and would generally require more immediate medical evaluation and intervention by a healthcare professional.

A change in severity of an adverse event will not be recorded as a new adverse event. Only the highest severity level that occurs during the entire period of the adverse event will be recorded on the CRF with the onset and resolution dates encompassing the entire duration of the event.

5.5 Assessing Causal Relationship (Relatedness)

For all adverse events, the investigator and the sponsor (the local medical monitor) will determine a **causal relationship,** to the study vaccine without knowledge, for blinded studies, of whether MVA85A/AERAS-485, placebo, or active control was administered. A number of factors will be considered in making this assessment, including: 1) the temporal relationship of the event to the administration of the study vaccine 2) whether an alternative etiology has been identified and 3) biological plausibility. The investigator will use the following guidelines to assess the causal relationship of an adverse event to study vaccine:

- **Not Related** to study vaccine (i.e., there is no evidence of a causal relationship; another etiology is known to have caused the adverse event. The alternative etiology should be documented in the infant's study record).
- **Unlikely Related** to study vaccine (i.e. there is less than a reasonable possibility that the adverse event was caused by study vaccine).
- **Possible** relationship to study vaccine (i.e., there is a reasonable possibility that the adverse event was caused by study vaccine. There must be a plausible mechanism for the event to be related to study vaccine. The evidence is inadequate to accept or reject, or favors rejection of, a causal relationship; an association exists between the event and the study vaccine but there may also be an alternative etiology, such as characteristics of the infant's clinical status or underlying condition).
- **Probable** relationship to study vaccine (i.e., it is likely that the adverse event was caused by administration of the study vaccine. The evidence favors acceptance of a causal relationship; an association exists between the event and receipt of the study vaccine and there is a plausible mechanism for the event to be related to the study vaccine, and an alternative etiology is not apparent).
- **Definite** relationship to study vaccine (i.e., the study vaccine is known to be the cause of the adverse event. The evidence establishes a causal relationship; an association exists between the event and receipt of the study vaccine and there is a plausible mechanism for the event to be related to the study vaccine, and causes other than the study vaccine have been ruled out).

The principal investigator and the local medical monitor both determine causality. It is expected that communication and consultation may occur in the assessment of the causality of adverse events. The greatest degree of causal relationship (definite > probable > possible > unlikely related > not related) determined by either the investigator or local medical monitor after their discussions will determine the ultimate classification of the adverse event. Definite, probable and possible are considered to be related. Not related and unlikely related are considered to be unrelated.

Every effort should be made by the investigator to determine the existence of any pre-existing conditions (e.g., mild nausea or headache on Study Day 0 with onset prior to study vaccination) that must be taken into consideration when assessing causal relationship of an adverse event. Pre-existing conditions should be recorded in the CRF as baseline history and substantiated by appropriate source documentation. Intermittent conditions such as headaches or menstrual pain may not be present on Study Day 0 but may represent an adverse event if the intensity or duration of the event is worse than usual following study vaccine.

5.6 Definition of Adverse Reaction

An adverse reaction is an adverse event judged to be related to study vaccine (see Section 5.3 for adverse event definition).

Related adverse events (adverse reactions) are defined as those judged by the investigator or local medical monitor to be possibly, probably, or definitely related to study vaccine.

The reporting period for all adverse events and adverse reactions is the 28-day post-vaccination follow-up period. Adverse events and adverse reactions will be reported on an Adverse Event CRF using a recognized medical term or diagnosis that accurately reflects the event.

5.7 Solicited Adverse Events and Injection Site Reactions

Solicited adverse events are events that a subject (or a subject's parents/guardians) is specifically asked about. These adverse events are commonly observed soon after receipt of vaccines. For this study, solicited adverse events include site of injection reactions, diarrhea, vomiting and others. Solicited adverse events of local injection site reactions will be considered causally related to study vaccine (adverse reaction).

The reporting period during which *solicited* adverse events will be obtained is the immediate 7 day period following each vaccination. The solicited adverse event reporting period begins with the day of vaccination. All infant's parents/guardians will be provided a diary card to record information regarding occurrences of these specific events. Solicited adverse events and temperature will be recorded by the infant's parents/guardians in a diary beginning on the day of vaccination through the specified time period. The diary card, when completed, will serve as source documentation for reporting on the Adverse Event CRF. Solicited adverse events will be entered on the Adverse Event Case Report Form.

Adverse events and solicited adverse events including assessment of local injection site reactions will be assessed by the investigator for severity, causal relationship to the study vaccine, possible etiologies, and whether the event meets criteria as a serious adverse event (and therefore requires immediate notification to the medical monitor).

Presence of ulceration and/or scarring at the site of injection and lymphadenopathy of the injection arm are considered to be adverse events that are causally related to the study vaccine and are of special interest. Site of injection ulceration (including presence of drainage) and axillary lymphadenopathy will be actively evaluated during each visit through the end of the study. These events will be recorded on the Adverse Event Case Report Form.

In the event that the clinical presentation meets the definition of a serious adverse event, in addition to the Adverse Event Case Report Form, a SAE Report must be completed and the event reported per protocol instructions.

5.8 Assessing "Seriousness" and Serious Adverse Events

Seriousness refers to the outcome of an adverse event. Seriousness is determined by both the principal investigator and the local medical monitor. If either principal investigator or local medical monitor determines an event to be serious, it will be classified as such. If any of the following outcomes are present then the adverse event is serious:

- It results in **death** (i.e., the AE caused or led to the fatality). Serious does not describe an event which hypothetically might have caused death if it were more severe.
- It was immediately **life-threatening** (i.e., the AE placed the subject at immediate risk of dying. It does not refer to an event which hypothetically may have led to death if it were more severe).
- It required inpatient **hospitalization** or prolonged hospitalization beyond the expected length of stay. Hospitalizations for scheduled treatments and elective medical/surgical procedures related to a pre-existing condition that did not increase in severity or frequency following receipt of study vaccine, are **not** serious by this criterion. Hospitalization is defined as a hospital admission or an emergency room visit for a period greater than 24 hours.
- It resulted in a persistent or significant **disability/incapacity** (i.e., substantial reduction of the subject's ability to carry out activities of daily living).
- It resulted in a **congenital anomaly or birth defect** (i.e., an adverse finding in a child or fetus of a subject exposed to the study vaccine prior to conception or during pregnancy).
- Other **medically important conditions** that may not result in death, threaten life or require hospitalization (i.e., the AE does not meet any of the above serious criteria) may be considered a serious adverse event when, based on appropriate medical judgment, they may jeopardize the subject and require medical or surgical intervention to prevent one of the serious outcomes listed in these criteria (e.g., allergic bronchospasm requiring intensive treatment in an emergency room or at home; blood dyscrasias or convulsions that do not result in hospitalization, or the development of drug dependency or drug abuse).

A **serious adverse event** is an adverse event meeting the outcome criteria for seriousness regardless of relationship to an administered medicinal product.

5.9 Assessing Expectedness

Expected adverse events are adverse events consistent with the applicable product information provided by the sponsor (the investigator's brochure for an investigational product). The sponsor, in the person of the local medical monitor, determines expectedness. If the assessment is that the adverse event is **expected** no further action is required. If the local medical monitor's assessment is that the adverse event is **unexpected,** then the event may represent a SUSAR (see Sections 5.10 and 5.11).

5.10 Definition of Suspected Unexpected Serious Adverse Reaction (SUSAR)

When an adverse event is judged to be related to an investigational product and also is judged to be serious and unexpected, it is a SUSAR (suspected unexpected serious adverse reaction) and is subject to expedited reporting.

5.11 Reporting of Serious Adverse Events

***Serious adverse events,* which include SUSARs, are reported to the sponsor and to the World Wide Safety Center (administered by PPD, Inc.) for the entire study period (see protocol appendices). SUSARs are reported even after the trial is over, if the sponsor, local medical monitor or principal investigator become aware of them.** The site will be provided with specific reporting procedures including reporting forms to be used. Serious adverse events will be reported using a recognized medical term or diagnosis that accurately reflects the event.

Serious adverse events will be assessed by the investigator and the local medical monitor according to their roles (as described in Sections 5.1.2 and 5.1.4) for severity, causal relationship to the study vaccine, and expectedness. The onset and resolution dates of the event and the action taken in response to the event will be documented. If the event has not resolved by the final study visit, it will be documented as "ongoing" on the CRF, however, follow-up of the SAE must continue until resolved. Information recorded on the CRF must be substantiated in the source documents.

An SAE Report completed for that event must be faxed or scanned and emailed by the principal investigator or his/her designee within 24 hours (one calendar day) of the investigative site becoming aware of the event to the local medical monitor, to the World Wide Safety Center and to the CRO, Triclinium . The SAE Report (paper form) should be completed and faxed (even if all information concerning the event is not yet known) within the first 24 hours of awareness of the event.

Fatal or life-threatening serious adverse events that the investigator suspects are related to the study vaccine should be telephoned to the local medical monitor immediately upon the investigator's awareness of the event. If the local medical monitor is required by the protocol or chooses to suspend enrollment s/he shall immediately create a written memorandum for record to the study file and telephonically notify the sponsor of this act.

Contact information for all safety personnel are contained in the Team Contact List which will be stored on site in the Site Regulatory Binder and maintained by the study sponsor.

Investigators must not wait to collect additional information to fully document the event before notifying the local and global medical monitors of a serious adverse event. The initial notification should include the following (at minimum):

- Protocol number and name and contact number of the investigator
- Subject ID number (and initials and date of birth, if available)

- Date subject received study vaccine
- Serious adverse event(s) and date of event onset
- Current status of subject

Aeras has authorized the PPD World Wide Safety Center to execute its responsibilities for safety report submission to the appropriate regulatory authorities within specific time periods of being notified of the event (within 7 or 15 calendar days depending the character of the SUSAR); therefore, it is important that the investigator submit additional information requested as soon as it becomes available. Triclinium will notify MCC; SATVI will notify UCT REC.

Aeras will notify the investigators at Oxford University and the Safety Monitoring Committee of all SAEs within three working days of becoming aware of an event and will provide all follow-up information in a timely manner.

5.12 Other Events Requiring Immediate Reporting

The investigator must report the following events by faxing the appropriate form to the local medical monitor and Triclinium within 24 hours of becoming aware of the event:

- Withdrawal of consent during the study (Immediately Reportable Event Form)
- Emergency unblinding (Immediately Reportable Event Form)
- Protocol violation affecting the safety of a subject or involving the vaccination process (Immediately Reportable Event Form)
- Adverse event thought to be an allergic reaction to the study vaccine (Immediately Reportable Event Form, unless event meets SAE criteria)
- Any event that, in the opinion of the investigator, precludes further administration of the study vaccine (Immediately Reportable Event Form, unless meets SAE criteria)

5.13 Adverse Event Treatment, Follow-up and Outcome

Treatment of any adverse events will be determined by the investigator using his/her best medical judgment and according to current clinical practice guidelines. All applied measures as well as follow-up will be recorded in the appropriate CRF.

Adverse events will be considered resolved when the condition returns to normal or returns to the subject's baseline status as established on Study Day 0, or when the condition has stabilized with the expectation that it will remain chronic.

The investigator will continue follow-up on adverse events, including laboratory abnormalities and solicited adverse events, until the event has resolved, is otherwise satisfactorily explained, or the subject completes the study. If not reported earlier, the outcome of adverse events and solicited adverse events will be determined at the end of the specified post-vaccination adverse event reporting period.

Follow-up for serious adverse events must continue until resolution and the outcome reported to Aeras, even if this extends beyond the serious adverse event reporting period (i.e., after the final study visit). For analysis purposes, the outcome for serious adverse events will be determined on the final study visit.

Outcome of all adverse events will be classified as one of the following:

- Resolved
- Resolved with sequelae
- Ongoing
- Death

If at any time after completion of the serious adverse event reporting period (the final study visit) the investigator becomes aware of a serious adverse event that is suspected by the investigator to be related to the study vaccine, the event must be reported to Aeras.

5.14 Long-term Safety Surveillance

Subjects enrolled in all Aeras clinical protocols who receive study vaccine will be enrolled into a vaccine development registry protocol for long term surveillance. An informed consent for this protocol will be signed by all enrolled in Aeras clinical product protocols. The primary objective of the registry is to collect cumulative serious adverse event experience and pregnancy outcomes from all clinical trial subjects who have entered an MVA85A/AERAS-485 clinical development study (or other eligible study) until at least two years after marketing authorization has been granted or until the product has been withdrawn from development.

5.15 Subject Diary and Daily Temperature Monitoring

Parents/guardians of infants who receive study vaccine will receive, and be instructed in, the operation of a daily adverse event diary, a thermometer and a ruler to be used during the specified post-vaccination diary period after vaccine administration. The daily adverse event diary is a source document and will record solicited adverse events, daily thermometer assessments of body temperature, and any other adverse events. During scheduled visits through the specified diary period after study vaccine administration the daily diary will be collected and reviewed by the principal investigator (or designee) at which time any clinical details required for complete understanding of the information recorded will be obtained. Diaries not brought to the scheduled visit should be obtained before adverse event assessment can be performed. Lost diaries will be reconstructed as possible on a new diary booklet by the infant's parents/guardian from memory on the closest scheduled visit and labeled as a reconstructed diary. Adverse events obtained from the diary will be recorded and completely assessed on the adverse event case report form. Body temperatures below 38º C will not be considered fevers.

Any change to an AE recorded by the infant's parents/guardian on the diary card (e.g., the severity level of an event is changed after interviewing the infant's parents/guardian) based on the investigator's evaluation of the event must be explained by notation in source documentation.

6 STOPPING RULES

The rules for pausing study vaccine administration by the principal investigator and local medical monitor are in Section 5.1. These rules govern the stopping of further study vaccine administration at any time during the study.

The rules for stopping further enrollment and study vaccine administration by the SMC are below:

- Death in any infant unless the SMC determines it is UNRELATED to MVA85A/AERAS-485
- An anaphylactic reaction to MVA85A/AERAS-485 in any infant
- The occurrence of a serious adverse event unless the SMC determines it is UNRELATED to MVA85A/AERAS-485.

The SMC may permit resumption of study vaccine administration if the study pause was for reasons less severe than those in the SMC stopping rules. The SMC may permit resumption of enrollment if it judges that changes to the study protocol will eliminate or greatly reduce the safety risks specified in the stopping rules. In the absence of study protocol changes, the SMC must follow the SMC study stopping rules.

If a decision to resume study enrollment and study vaccine administration is made the SMC will record their judgment in a memorandum to the study file and notify the sponsor, who will then forward the SMC memorandum to the medical monitors and principal investigators. The clinical site will be allowed to resume activities upon receipt of written notification from the sponsor. The appropriate regulatory authority will be informed in writing if the study is stopped and will be informed in writing of the decision by the SMC to resume or discontinue study activities.

7 STATISTICAL CONSIDERATIONS

The planned statistical analyses for this study are outlined below. A detailed statistical analysis plan for preparation of the final study report will be created and made final prior to database lock and before the study is unblinded. A separate analysis plan will be prepared in conjunction with the planned SMC reviews of blinded and unblinded-by-group safety data through Study Day 84 for the safety cohort (Study Group 1) and for the cumulative safety review following completion of the Study Day 84 visit by the 1000th enrollee (see Section 5.1.5), and in conjunction with the interim reviews of accrued TB cases (see Section 7.6); these analyses will be conducted by an unblinded statistician not associated with the study conduct or primary analyses. Separate statistical analysis plans will also be prepared for the gene expression analysis and in conjunction with any summarization of immunogenicity results to be prepared prior to unblinding for preparation of the final study report.

7.1 Subject Populations

The safety population will consist of all subjects who received a dose of study vaccine. The per-protocol population for efficacy analyses will consist of all randomized subjects enrolled

in the study who received a dose of study vaccine and had no major protocol deviations. The intent-to-treat population for efficacy analyses will consist of all randomized subjects.

7.2 Demographics and Protocol Compliance

Demographic parameters and other baseline characteristics (age [days] at Study Day 0, sex, and race/ethnicity) will be summarized by treatment group, study group, and for all subjects in the safety population. As subject enrollment into individual study groups (cohorts) will be determined based on time of completion of eligibility, any imbalance in baseline characteristics across study groups will be examined.

Listings of randomized subjects who did not receive study vaccine and of subjects with protocol deviations (to be defined in the statistical analysis plan) will be provided.

7.3 Efficacy Analyses

Although this study is not designed as an efficacy study, subjects in all study groups will be followed for the development of tuberculosis. Preliminary analyses to evaluate the secondary objective of the efficacy of MVA85A/AERAS-402 will be based on the per-protocol population, as defined in Section 7.1. A secondary analysis will be conducted based on the intent-to-treat population. Clinically applicable definitions of TB disease for this study population will be investigated in this proof-of-concept study.

7.3.1 Case definition of tuberculosis

Subjects will be evaluated for the development of TB disease throughout the study based on the procedures described above. Two sets of case definitions for TB will be evaluated in this proof-of-concept study and analyzed in conjunction with the secondary objective of the efficacy of MVA85A/AERAS-485 compared with control. Subjects will be evaluated for the secondary endpoint of TB disease using the clinically relevant TB case definitions below (TB Case Definition Endpoint #1 and #2) as described below. An endpoint related to treatment (TB Case Definition Endpoint #3) will also be analyzed.

The TB case definition endpoints will be applied and analyzed as described below both for subjects investigated for TB in the study case verification ward, and for those subjects who receive a clinical diagnosis of TB disease as part of routine care in other health facilities for whom hospital record review demonstrates that the case meets the study case definition of TB disease given possible limitations in the quality and completeness of all relevant assessments. Handling of missing data during application of the TB Case Definition (e.g., missing chest x-ray data) will be detailed in the statistical analysis plan for the final study report.

TB Case Definition Endpoint #1
Any of the following numerical categories

1. Isolation of *M. tuberculosis* from any site.
2. Identification of *M. tuberculosis* by an approved molecular diagnostic technique from any site.
3. Histopathology diagnostic for tuberculosis disease (such as caseating granulomas)

4. Choroidal tubercle diagnosed by an ophthalmologist
5. Miliary pattern on chest X ray in a HIV negative infant
6. Clinical diagnosis of tuberculous meningitis (CSF protein >0.6 g/L and pleocytosis >50/mm^3 with mononuclear cell >50%) with features of basal meningeal enhancement and hydrocephalus on head CT.
7. Vertebral spondylosis
8. A single smear/histology specimen positive for acid fast (or auramine positive) bacilli from a normally sterile body site.
9. One of the following:
   a) Two acid fast or auramine smears positive each from a separate collection morphologically consistent with mycobacteria from either sputum or gastric aspirate that are not found to be non-tuberculous mycobacteria bacteria on culture , OR
   b) QuantiFERON conversion from negative or indeterminate to positive, OR
   c) Tuberculin skin test ≥15 mm

   AND

   One of the following compatible radiographic features:
   a) Calcified Ghon focus, OR
   b) Pulmonary cavity, OR
   c) Hilar/mediastinal adenopathy, OR
   d) Pleural effusion, OR
   e) Airspace opacification,

   AND

   One of the following clinical manifestations:
   a) Cough without improvement for longer than two weeks, OR
   b) Weight loss of at least 10% of body weight for at least 2 months, OR
   c) Failure to thrive (crossing at least one entire major centile band downward) for at least 2 months, where the major centile bands are defined as <97th–90th, <90th–75th, <75th–50th, <50th–25th, <25th–10th, and <10th–3rd weight-for-age centiles*.
   **Centers for Disease Control (CDC) Growth Charts (USA), developed by the National Center for Health Statistics in collaboration with the National Center for Chronic Disease Prevention and Health Promotion (2000).*

TB Case Definition Endpoint #2

Any of the following numerical categories

1. Isolation of *M. tuberculosis* from any site.
2. Identification of *M. tuberculosis* by an approved molecular diagnostic technique from any site.
3. Histopathology diagnostic for tuberculosis disease (such as caseating granulomas)
4. Choroidal tubercle diagnosed by an ophthalmologist.
5. Miliary pattern on chest X ray in a HIV negative infant

6. Clinical diagnosis of tuberculous meningitis (CSF protein >0.6 g/L and pleocytosis >50/mm^3 with mononuclear cell >50%) or features of basal meningeal enhancement and hydrocephalus on head CT.
7. Vertebral spondylosis
8. A single smear/histology specimen positive for acid fast (or auramine positive) bacilli from a normally sterile body site.
9. One of the following:
   a) One acid fast or auramine smear positive morphologically consistent with mycobacteria from either sputum or gastric aspirate that are not found to be non-tuberculous mycobacteria bacteria on culture , OR
   b) QuantiFERON conversion from negative or indeterminate to positive, OR
   c) Residence in a household with an AFB smear positive member, OR
   d) Tuberculin skin test ≥10 mm

   AND

   One of the following compatible radiographic features:
   a) calcified Ghon focus, OR
   b) pulmonary cavity, OR
   c) hilar/mediastinal adenopathy, OR
   d) pleural effusion, OR
   e) airspace opacification, OR

   AND

   One of the following clinical manifestations:
   a) Cough without improvement for longer than two weeks, OR
   b) Weight loss of at least 10% of body weight for at least 2 months, OR
   c) Failure to thrive (crossing at least one entire major centile band downward) for at least 2 months, where the major centile bands are defined as <97th–90th, <90th–75th, <75th–50th, <50th–25th, <25th–10th, and <10th–3rd weight-for-age centiles*.
   **Centers for Disease Control (CDC) Growth Charts (USA), developed by the National Center for Health Statistics in collaboration with the National Center for Chronic Disease Prevention and Health Promotion (2000).*

TB Case Definition Endpoint #3
All individuals who are placed on anti-tuberculosis therapy with the intent of treating tuberculosis regardless of whether they have met the other efficacy endpoints

7.3.2 Incidence of TB disease

The number (percentage) of subjects with a diagnosis of TB disease, as defined by TB Case Definition Endpoint #1 in Section 7.3.1, will be used to evaluate the secondary objective of the efficacy of the MVA85A/AERAS-485 vaccine compared to controls in prevention of tuberculosis. The number of subjects with a diagnosis of TB disease will be summarized by treatment group, study cohort, and for all subjects in the per-protocol population. Additional

analyses will be performed based on the intent-to-treat population, and for all subjects who complete the study through end of protocol follow-up. The cumulative person-years incidence of TB and associated 95% confidence intervals during study follow-up will be summarized by treatment group and by study group. Person-time observation will be used to estimate the time at risk of TB and will be calculated based on subject date of last contact with the study minus the date of Study Day 0 vaccination. The incidence of TB disease will be calculated as the number of new cases of TB disease diagnosed during study follow-up, divided by the total person-time of observation. Comparison of the incidence of TB in each treatment group will be performed using relative risk summaries and corresponding 95% confidence intervals, as appropriate, for all subjects in the per-protocol population. Vaccine efficacy for MVA85A/AERAS-485 will be calculated with corresponding 95% confidence interval as (1 – relative risk) x 100. Subjects with multiple TB cases or recurrence will only be counted once in summaries of cumulative incidence.

Time to initial TB diagnosis using Case Definition Endpoint #1 from Study Day 0 in each treatment group will be examined using the Kaplan-Meier estimate of the survival function, and compared using the log-rank test or alternative methodology based on distributional assumptions (to be addressed in the statistical analysis plan). Median time to initial diagnosis and associated 95% CI will be presented by treatment group and for all subjects in the per-protocol population.

The number (percentage) of subjects with a diagnosis of TB disease as defined by TB Case Definition Endpoint #2, and the number (percentage) of subjects meeting either TB Case Definition Endpoint (#1 or #2, Section 7.3.1) will also be summarized by treatment group, study cohort, and for all subjects in the per-protocol population and will be analyzed as described for TB Case Definition Endpoint #1 above. Sensitivity analyses based on the intent-to-treat population will also be performed. Additional analyses will examine differences in number (percentage) of subjects and time to diagnosis for subjects who are excluded from either specific diagnostic criteria categories, and for underlying criteria categorizations (e.g., TST measurement differences).

Exploratory analyses of the number (percentage) of subjects who are placed on anti-tuberculosis therapy during the study for the purpose of treating TB (TB Case Definition #3) will also be performed. Subjects who meet the criteria for this exploratory analysis may or may not also be included in the analyses of the primary TB diagnostic criteria given the endpoint case definitions as defined above. Number (percentage) of subjects placed on anti-TB treatment will be summarized by treatment group, study cohort, and for all subjects, and will be analyzed as described for the TB Case Definition Endpoints #1 or #2, above. Additional sensitivity analyses will be performed to examine differences in number (percentage) of subjects and time to diagnosis for those subjects who are prescribed TB treatment but who do not meet criteria for TB Case Definition Endpoints #1 or #2. Additional exploratory analyses of subjects receiving isoniazid prophylaxis as a result of, e.g., close contact with an adult diagnosed with tuberculosis, will also be performed if warranted by the number of cases. Number (percentage) of subjects receiving isoniazid prophylaxis will be summarized by treatment group and study cohort, with additional analyses performed as described for the TB Case Definition Endpoints #1 or #2, above.

7.4 Immunogenicity Analyses

The primary immunogenicity analyses will be based upon the subjects who received at least one dose of study vaccine and were enrolled in one of the three immunogenicity cohorts. No imputation for missing data will be performed. Data will be transformed as appropriate prior to analysis.

7.4.1 Intracellular Cytokine Staining Assay

The primary variables of interest for assessment of immune response to MVA85A/AERAS-485 will be the percentage of CD4 and CD8 T cells that produce any one of three cytokines (IFN-γ, TNF-α, or IL-2) or any combination of the three cytokines simultaneously following stimulation with peptide pools containing the entire amino acid sequence of Ag85A. Response will be measured by flow cytometry in the intracellular cytokine staining (ICS) assay.

Descriptive statistics will be used to summarize percentage CD4 and CD8 T cell response by treatment group and stimulation antigen (Ag85A), for all subjects in the ICS immunogenicity cohort. Summaries will include the assessment of response at all pre- and post-vaccination immunology time points, as well as change from pre-vaccination to each post-vaccination time point. MVA85A/AERAS-485 immune response will be compared with placebo across all post-immunization time points using a linear model. The model will include time, treatment group, and appropriate interaction terms, and selection of an appropriate covariance structure for the multiple response measures. If T cell response is not normally distributed, appropriate transformations or an alternative model (e.g., area under the curve (AUC) as the measure of immune response) will be explored. Individual models will be prepared by stimulation antigen for CD4 and CD8 T cell response.

The area under the curve (AUC) and associated 95% confidence interval will be used to summarize the immune response by treatment group. Area under the curve will be calculated using the trapezoidal rule, from Study Day 0 across all available post-vaccination time points. An exploratory comparison of the mean AUC for MVA85A/AERAS-485 and control will be performed using the two-sample test.

7.4.2 ELISPOT

An *ex vivo* IFNγ ELISPOT assay will be used to assess specific T cell responses to antigen 85A and other mycobacterial antigens. Descriptive statistics will be used to summarize T cell responses by treatment group and stimulation antigen, for all subjects in the Elispot immunogenicity cohort. Summaries will include the assessment of response at all pre- and post-vaccination immunology time points, as well as change from pre-vaccination to each post-vaccination time point. Additional analyses as described for the ICS assay in Section 7.4.1 may also be presented.

7.4.3 Whole Blood Assay

Concentrations of Th-1 (including IFN-gamma) and Th-2 cytokines will be obtained from supernatants of whole blood cultures stimulated with mycobacterial antigens, for all subjects in the whole blood assay cohort. Cytokine concentrations will be determined with standardized quantitative methods such as ELISA or Multiplex. Cytokine levels at pre and post vaccination time points will be calculated by applying a cytokine standard. Results will be summarized using descriptive statistics at each time point. Additional analyses as described for the ICS assay in Section 7.4.1 may also be presented.

7.4.4 Correlates of Protection

Investigations for determining correlates of immune protection to tuberculosis are ongoing and the technical specifications for the assays are unknown at this time. Decisions on the technical aspects for completing the analysis of correlates of immune protection for this study will be made before the study is unblinded. Further information on the planned analysis will be provided in a separate analytic plan, when available.

7.4.5 QuantiFERON conversion rate

QuantiFERON®-TB Gold test results at Study Day 0, at Study Day 336 and at the last clinic visit, and will be summarized by number (percentage) of positive responses by treatment group and by study group. Cross-tabulation of the number of subjects with a negative QuantiFERON with subsequent conversion to a positive result post vaccination will be summarized by treatment group and by study group. The number (percentage) of subjects with on-study QuantiFERON conversion will also be summarized for those with and without a diagnosis of TB disease during the study, by time point, treatment group, study group, and for all subjects in the safety population.

7.4.6 Gene Expression

The innate immune response to vaccination will be examined as an exploratory objective in this study. A sequentially-enrolled subset of up to 100 randomized subjects will be analyzed using gene expression and transcriptional profile of blood collected at Study Day 1 post-vaccination. Immune response for these subjects will be analyzed using microarray analysis. Analytic techniques will be summarized in a separate analysis plan. The sample size of 100 infants has been selected for an initial review of immune response using these techniques, rather than for statistical reasons. Enrollment into this subset will be performed across study groups, with timing of enrollment contingent on order of completion of screening and consent for this additional evaluation; as a result, the number of subjects in the subset may not be equally allocated between MVA85A/AERAS-485 and control groups.

7.5 Safety Analyses

Safety analyses will be performed using the safety population as defined in Section 7.1.

Summaries of adverse events will be presented for all subjects. Analysis of clinical laboratory results and unblinded safety review following completion of the Study Day 84 time point will be presented based on the study vaccine Safety Cohort (Study Group 1).

7.5.1 Adverse Events

The primary variable for evaluation of the safety of MVA85A/AERAS-485 will be the number and percentage of solicited and unsolicited adverse events (including SAEs) recorded post-vaccination, by MedDRA system organ class (SOC), preferred term, study group, and treatment group.

The number (percentage) of subjects with adverse events occurring within the post-vaccination adverse event reporting period will also be summarized. Additional summaries will include presentations by adverse event reporting period, by severity, and by relationship to study vaccine. Each subject will be counted once per term at the greatest severity or most related state recorded for that term.

Separate summaries for the number (percentage) of subjects with solicited adverse events (injection site reactions and general symptoms) will also be presented. Solicited adverse events will also be summarized by severity; each subject will be counted once per term at the greatest severity recorded for that term.

Serious adverse events (SAEs) will be recorded through the end of study for all subjects. Listings will be provided for all subjects with serious adverse events. Listings will also be provided for all subjects who have discontinued prematurely due to an adverse event.

The number (percentage) of subjects in the Safety cohort with post-vaccination clinical laboratory values recorded as newly abnormal following study vaccination and meeting toxicity mild criteria (Grade 1) or above as specified in the Aeras Pediatric Toxicity Table (Appendix C) will be tabulated at each post-vaccination time point and overall. Clinical laboratory abnormalities will also be reported as adverse events and will be included in the summary of adverse events.

7.5.2 Other Safety Assessments

For clinical laboratory parameters and vital signs assessments pre-specified in the protocol, summary statistics for continuous parameters will be presented for Day 0 pre- and post-vaccination time points, and for the change from pre-vaccination to post-vaccination assessment, by treatment group and study group.

7.6 Sample Size Considerations

The sample sizes were selected because they are judged to be adequate for safety evaluations. A sample size of 1392 MVA85A/AERAS-485 subjects will give a greater than 75% chance of observing an adverse event that has an approximately 1 in a 1000 actual rate of occurrence. A sample size of 165 MVA85A/AERAS-485 subjects in the safety cohort will provide an 80% chance of observing at least one adverse event which has an approximate 1% actual rate of

occurrence. Given a tuberculosis cumulative incidence of 3% over 18 months in the control group, 1392 subjects per treatment group (2784 subjects total) would be required to demonstrate positive efficacy when the true efficacy of MVA85A/AERAS-485 is approximately 60%. An estimate of 7.5% of subjects lost to follow-up in each treatment group is assumed over 18 months. Calculations are based on a two-sided log-rank test at a significance level of 0.05. Calculations assume 90% power to detect a 60% reduction in tuberculosis rate between the MVA85A/AERAS-485 and control groups; given an estimated enrollment over 21 months, the last subject to enroll in the study will be followed for a minimum of 15 months to maintain statistical power.

However, delays in recruitment and/or a lower TB incidence may result in a need to extend follow-up to preserve the power of the study to detect a treatment group difference. To assess the possible impact of departures from planned subject recruitment rate, total number of TB cases accrued, loss to follow-up, or proportional hazards assumptions, an independent statistician not involved with the conduct or analyses of the trial will perform a sample size review using an interim pooled estimate of the TB event rate and the original treatment effect. It is critical that this interim review occur when sufficient TB events have accrued, to allow for a stable estimate, but before the total cohort has been recruited, due to trial management issues. The initial sample size review is proposed to occur while enrollment is ongoing but when approximately 30% of expected events have accrued, but no later than before 75 percent of the total cohort has been recruited. Details of this review and analysis will be documented in a statistical analysis plan, finalized prior to the review, and in the SMC charter. Timing of subsequent reviews will be determined based on planned completion of recruitment closure after 21 months and enrollment rate.

These reviews will not result in a statistical penalty as no treatment comparisons will be performed. The monitoring of these parameters and possible courses of action will be detailed in a separate statistical analysis plan and in the SMC Charter. Further reviews and/or unblinding by the SMC to treatment assignments will be performed at the discretion of the SMC if, based on their judgment, additional information is warranted to better assess ongoing event rates to achieve the endpoint of the study.

The SMC mandate, oversight schedule, and membership will be delineated in the SMC Charter. The statistical analysis plans to be prepared for all analyses will be approved by the SMC. Aeras and study site personnel will remain blinded to treatment assignments and interim analyses by-treatment results throughout the study.

7.7 Interim Immunogenicity Review

The sponsor may conduct a review of immune response data prior to database lock after immunogenicity results are available for each of Study Groups 2, 3 and 4. Each Study Group will be analyzed individually. Blinded data will be available to investigators. Aggregate data will be presented at selected time points and will be presented identified only by a blinded label (e.g., ‘Group A’ and ‘Group B’).

Review of these data will allow the sponsor to determine if there is evidence of any immune response prior to completion of the trial. No decision cut points or stopping rules will be stipulated, no changes to monitoring practices or study procedures will be implemented following the reviews, and the study will continue following the reviews. No hypothesis testing will be performed.

Data summaries for preliminary immunogenicity review using blinded labels will be prepared by a statistician who is not the study statistician and who is not involved in study design, conduct, or analyses. Immunology technicians will perform all assays in a blinded manner. Data identified for inclusion in the immunogenicity summaries will be cleaned prior to statistical analysis; all statistical analysis programs used for the study will be validated prior to presentation of results.

7.8 Safety Reviews

A blinded and unblinded-by-group review of safety data will be prepared for the SMC when the last subject in the Safety Cohort (Study Group 1) completes Study Day 84, and when the 1000th enrollee completes Study Day 84 as described in Section 5.1.5 and in the SMC Charter. Both blinded and unblinded analyses will be described in a statistical analysis plan, and unblinded reviews will be prepared for the SMC by an unblinded statistician not associated with the study conduct or primary analyses. All procedures associated with this review, including objectives, data handling, and elements to be included for review will be documented in the SMC minutes.

7.9 Final Study Report

A final study report will be prepared after database lock is completed for all data collected through the final assessment of the study.

7.10 Computer Methods

Statistical analyses will be performed using SAS® version 9.1 or later under a Windows operating system.

8 DATA COLLECTION, MONITORING, AND RECORD RETENTION

For the purpose of monitoring and auditing the study, source documentation will consist of existing medical records and/or study records developed and maintained by the investigator. Any source document templates provided by Aeras or its designee will serve as supplements to the subject's study record.

Data recorded on source documents will be transcribed onto case report forms (CRF) provided by Aeras, or entered using electronic case report forms (eCRFs) using an Electronic Data Capture (EDC) system provided and approved by Aeras. Completed, original CRF will be retrieved by Aeras or its designee and a copy of each completed CRF (as applicable) will be retained at the clinical site as part of the study records.

The study will be monitored regularly by Aeras or its designee throughout the study period. For studies of unapproved investigational products, all study records (source documents, signed informed consent forms, copies of CRF (as applicable), IRB/IEC correspondence and approval letters, study vaccine management records) will be kept secured for a minimum of two years following the marketing of the investigational product or for 2 years after the discontinuation of the IND (or CTA, etc.). The investigator will ensure that study records are not disposed of or removed from the clinical site without prior notification and approval from Aeras or its designee.

9 HUMAN SUBJECTS

9.1 Ethics and Regulatory Considerations

The study will be conducted according to the Declaration of Helsinki, ICH-GCP, Protection of Human Subjects (21 CFR 50), Institutional Review Boards (21 CFR 56), Obligations of Clinical Investigators (21 CFR 312), and local regulatory requirements.

The protocol and informed consent form will be reviewed and approved by the IRB or IEC of each participating clinical site prior to any protocol-specified procedures being conducted. The investigator will inform the IRB/IEC as to the progress of the study on a regular basis, or at minimum, once a year. Aeras will also have an independent IRB review and approval of the protocol and informed consent form and will keep the IRB informed of the progress of the study.

Written informed consent will be obtained from each parent/guardian prior to any protocol-specified procedures being conducted.

To maintain confidentiality, subject identification numbers will be used to identify the subject's laboratory specimens, source documents, CRF, study reports, etc. All study records will be maintained in a secured location. Clinical information will not be released without written permission from the subject's parent/guardian except as necessary for monitoring or auditing of the study by Aeras or its designee or applicable regulatory authorities.

9.2 Institutional Review Board or Independent Ethics Committee

All the documents the IRB/IEC may need to fulfill its responsibilities, such as the protocol, protocol amendments, information concerning subject recruitment, payment or compensation procedures, etc., will be submitted to the IRB/IEC by the investigator. The IRB's/IEC's written, unconditional approval of the study protocol and the informed consent form will be in the possession of the investigator/clinical site staff prior to the conduct of any protocol-specified procedures.

Modifications to the protocol may not be implemented without prior written IRB/IEC approval except when necessary to eliminate immediate hazards to the subjects or when the modification involves only logistical or administrative aspects of the study. Such logistical or

administrative modifications will be submitted to the IRB/IEC in writing by the investigator, and a copy of the correspondence to verify the submission will be maintained.

The investigator must inform the IRB/IEC of modifications to the informed consent or any other documents previously submitted for review/approval, of any new information that may adversely affect the safety of the subjects or the conduct of the study, provide an annual update and/or request for re-approval, and advise the IRB/IEC when the study has been completed.

Any documents or forms to be provided to the subject (e.g., information cards, form letters from the investigator), and all forms of study advertising (flyers, brochures, print advertisements, radio or television scripts, etc.) must be approved by Aeras or its designee prior to the clinical site submitting them to the IRB/IEC. Approval from the IRB/IEC must be obtained prior to providing the documents or forms to the subject.

9.3 Informed Consent

The principles of informed consent in the current edition of the Declaration of Helsinki and 21 CFR 50.25 should be implemented prior to any protocol-specified procedures being conducted.

Informed consent will be documented in writing on a consent form approved by the IRB/IEC.

All relevant information should be provided in both oral and written form in a way that is understandable to the subject's parent/guardian. Ample time and opportunity must be given for the subject to inquire about details of the study. The written consent document will embody the elements of informed consent as described in the Declaration of Helsinki and will also comply with local regulations.

The investigator or the investigator's qualified designee will explain the nature of the study and inform the subject that participation is voluntary and that the subject can withdraw at any time.

The subject must be informed about the study's purpose including why the subject was selected to participate, study goals, expected benefits and risks, potential risks, and that some potential risks are unforeseeable. The individual must be provided with a description of the procedures and the estimated duration of time required for participation in the study, as well as alternative interventions or courses of treatment, if applicable.

The subject must receive an explanation as to whether any compensation and any medical treatments are available if injury occurs and, if so, what they are, where further information may be obtained, and who to contact in the event of a study-related injury. Individuals must be told who to contact for answers to any questions related to the study.

The subject must be informed that their participation is voluntary and that they are free to withdraw from the study for any reason at any time without penalty or loss of benefits to

which they are otherwise entitled. The extent of the confidentiality of subject records must be defined and the subject must be informed that applicable data protection legislation will applies.

The subject must be informed that the monitor(s), auditor(s), IRB/IEC members, and the applicable regulatory authorities will be granted direct access to the subject's original study medical records for verification of protocol-specified procedures and/or data, without violating the confidentiality of the subject to the extent permitted by the applicable laws and regulations. The subject must be informed that his/her signature on the informed consent form indicates that he/she has decided to participate in the study, having read and discussed the information presented.

Modifications made by the investigator to an informed consent form template provided to the investigator by Aeras or its designee will be reviewed and approved by Aeras or its designee prior to being submitted to the IRB/IEC.

The original, signed informed consent form for each subject will be maintained by the investigator as part of the subject's study records. A copy of the signed informed consent form will be provided to each subject.

10 STUDY COMPLETION

At the discretion of Aeras, all materials and supplies provided to the investigator will be returned or disposed of in compliance with local regulatory requirements upon authorization from Aeras, upon study completion. The investigator or designated clinical site staff will notify the IRB/IEC when the study has been completed.

11 PUBLICATIONS

The final study report submitted to regulatory authorities will be made available to the principal investigator for purposes of publications. The investigator and clinical site staff must send all manuscripts, abstracts, and presentations using data from this study to Aeras for review prior to their submission. Aeras reserves the right to delete any part or parts of such materials deemed to be confidential or proprietary.

12 CHANGES TO THE PROTOCOL

The protocol may not be modified without written approval from Aeras. All changes to the protocol must be submitted to the IRB/IEC and must be approved by the IRB/IEC prior to their implementation.

12.1 List of Changes from Protocol Version 1.0 to Version 2.0

The changes that were made to protocol version 1.0 (dated 21 May 2008) to create version 2.0 of the protocol are described below.

1. The principal investigators name and address have been added to the signature block on the Title Page.

2. Section 2.2, "Study Design," and the study synopsis
   Read: On Study Day 0, eligible infants will be sequentially assigned to a study group starting with Study Group 1 and randomly assigned within a study group to receive either MVA85A/AERAS-485 or *Candida* skin test antigen control…

   Changed to Read: On enrollment to the study, eligible infants will be sequentially assigned to a study group starting with Study Group 1 and, on Study Day 0, they will be randomly assigned within a study group to receive either MVA85A/AERAS-485 or *Candida* skin test antigen control…

   And
   Read: A QuantiFERON test will be drawn on Study Day 357 and on the last clinic visit in all infants without the clinical diagnosis of tuberculosis.

   Changed to Read: A QuantiFERON test will be drawn on Study Day 336 and on the last clinic visit in all infants without the clinical diagnosis of tuberculosis.

3. Section 3.4, "Study Vaccination," first sentence
   Read: Infants must receive their initial dose of study vaccine/control on Study Day 0 as soon as possible after randomization.

   Changed to Read: Infants must receive their dose of study vaccine/control on Study Day 0 as soon as possible after randomization.

4. Section 5.1.5, "Safety Monitoring Committee"
   Read: The SMC is composed of five voting members (two licensed physicians from South Africa, one licensed physician from the United Kingdom, and two licensed physicians from the US), a non-voting statistician, and an non-voting Aeras member who will record committee actions. At least two physicians with experience in evaluation of investigational product safety issues. The voting members can not be directly involved with the conduct of the study, can not be employees of Aeras and must be experienced in the evaluation of adverse events in clinical trials.

Changed to Read: The SMC is composed of five voting members (two licensed physicians from South Africa, one licensed physician from the United Kingdom, and two licensed physicians from the US) and may have up to two alternate physician members. The voting members can not be directly involved with the conduct of the study, can not be employees of Aeras and must be experienced in the evaluation of adverse events in clinical trials. The SMC will also have a non-voting statistician and a non-voting Aeras member who will record committee actions.

5. Section 8, "Data Collection, Monitoring, and Record Retention"
   Read: Data recorded on source documents will be transcribed onto case report forms (CRF) provided by Aeras.

   Changed to Read: Data recorded on source documents will be transcribed onto case report forms (CRF) provided by Aeras, or entered using electronic case report forms (eCRFs) using an Electronic Data Capture (EDC) system provided and approved by Aeras.

6. Appendix C, "Aeras Pediatric Toxicity Table," – The definitions of Grade 1 Erythema and Grade 1 Induration were corrected (i.e., "less than 1.0 cm in diameter" changed to "≤1.0 cm in diameter") as was the definition of Grade 4 Erythema, Induration and Ulceration.

7. Minor editorial changes and corrections of inconsistencies were also made.

12.2 List of Changes from Protocol Version 2.0 to Version 3.0

The changes that were made to protocol version 2.0 (dated 7 July 2008) to create version 3.0 of the protocol are described below.

Section Number (Title)	Version 2.0	Version 3.0
**Section** 3.2.3.1 ("Inclusion Criteria"), item #3	**Change/rationale:** The eligibility requirement for weight was changed to be consistent with local standards, which designate infants as underweight when they are at <3rd percentile.	
	Weight: by chart >10th percentile on Study Day 0	Weight: by chart >3rd percentile on Study Day 0
**Section** 3.2.3.1 ("Inclusion Criteria"), item #6	**Change/rationale:** The former requirement for 3 Prevenar injections prior to randomization were modified to be compatible with the immunization scheduled being planned by the South African Department of Health. Other references to three Prevenar injections prior to randomization were also removed throughout the protocol.	
	Must have completed three conjugated pneumococcal vaccine injections at least 28 day prior to Study Day 0	Must have received age-appropriate doses of pneumococcal vaccine as recommended by the South African Department of Health but no injection within 28 day prior to Study Day 0

Section Number (Title)	Version 2.0	Version 3.0
**Section** 2.2.1 ("Rationale for Blinding Procedures")	**Change/rationale:** Modified in recognition of the fact that Prevenar will be available through the South African Department of Health to eligible infants during the study. Other similar references were also removed or modified throughout the protocol.	
	Prevenar is a licensed vaccine that is not part of the standard vaccination recommendations but provides protection from pneumococcal disease in this age group and is required to be given to all those infants without contraindications who have completed a consent agreement for the study. Because of the standard vaccination schedule it was judged best to administer this benefit at the time of regular immunizations prior to Study Day 0.	Prevenar is a licensed vaccine and is required to be given to all those infants without contraindications who have completed a consent agreement for the study.
**Section** 2.0 ("Design") and the Study Abstract	**Change/rationale:** The original plan to enroll study groups sequentially has been modified to allow concurrent enrollment in Study Group 2-5 after Study Group 1 completes enrollment, because two clinics that will enroll infants do not have the facilities to handle the immunology specimens in Study Groups 2 and 4 and can therefore not enroll infants in these study groups. The new plan will facilitate enrollment and avoid the inconvenience of having infants complete screening procedures but not be able to enroll in an open study group.	
	Not present	Infants will be assigned to a safety cohort (Study Group 1), then into one of three immunological assay evaluation groups (Study Groups 2-4), and finally the remainder of infants will be assigned into the correlate of protection cohort (Study Group 5). At least 330 infants will be randomized in Study Group 1, 50-60 infants each in Study Groups 2-4, and the remaining infants will be randomized in Study Group 5 such that the total number of infants randomized on the study will be 2784.
**Section** 3 ("Study Procedures")	**Change/rationale:** The maximum ages associated with scheduled visits have been removed to allow flexibility in scheduling visits, but the minimum ages have been retained to insure blood collection is age-appropriate.	
	Throughout Tables 1-5	Throughout Tables 1-5
**Section** 3 ("Study Procedures")	**Change/rationale:** A urine collection has been added during the screening visits. The urine will be stored to evaluate possible future tests of TB diagnostics.	
	Throughout Tables 1-5	Throughout Tables 1-5
**Section** 3 ("Study Procedures")	**Change/rationale:** A footnote has been added to each table to clarify the timeframe for completing screening visits.	
	Throughout Tables 1-5	Throughout Tables 1-5
**Section** 3.3 ("Assignment to Study Group and Randomization")	**Change/rationale:** Has been modified to reflect the use of an interactive voice/web response system to enroll and randomize infants in the study	
	Following completion of screening and the three pneumococcal vaccine injections, infants will be sequentially enrolled into 1 of 5 Study Groups and, within each Study Group, infants will be	Infants will be randomized in a 1:1 ratio to MVA85A/AERAS-485 or control within each Study Group using an interactive voice/web response system (IVRS/IWRS). The randomization schedule will be prepared by a

Section Number (Title)	Version 2.0	Version 3.0
	randomized in a 1:1 ratio to MVA85A/AERAS-485 or control based on a randomly-generated sequence of subject identification numbers (randomization schedule). The randomization schedule will be prepared by a statistician who will not be involved with the analysis of the study in order to maintain blinding of the study team. Infants will be considered randomized when a subject identification number is assigned on Study Day 0.	statistician who will not be involved with the analysis of the study in order to maintain blinding of the study team.
**Section** 3.5.2 ("Clinical Assessments and Laboratory Tests")	**Change/rationale:** Modified to clarify that one of the immunologic assessments conducted in the TB case verification (CV) ward is a QuantiFERON (QFT-G).	
	All infants randomized to the study will have had blood for QFT-G will be collected during screening, again on Study Day 336 and on the last scheduled visit for each infant.	All infants randomized to the study will have blood for QFT-G collected during screening, again on Study Day 336, during evaluations for TB in the CV ward, and on the last scheduled visit for each infant.
**Section** 3.5.3 ("Immunology Laboratory Evaluations")	**Change/rationale:** Modified to allow for storage of cells/plasma for possible future testing to evaluate immune responses.	
	Not present	Plasma remaining from the immunologic assays in Study Groups 2-4 and from the cells collected in Study Group 1 and 5 will be stored frozen for possible future testing to evaluate immune responses.
**Section** 3.5.6 ("Diagnosis of Tuberculosis and Case Verification")	**Change/rationale:** Modified to clarify that one of the immunologic assessment conducted in the TB case verification (CV) ward is a QuantiFERON (QFT-G) and the addition of urine collection.	
	Blood will be drawn for HIV testing and other blood tests that are clinically indicated (e.g., CBC), as well as for tuberculosis immune assays.	At some time prior to discharge, urine will be collected and stored for possible future testing with Mtb diagnostic tests. Blood will be drawn for HIV testing and other blood tests that are clinically indicated (e.g., CBC), as well as for tuberculosis immune assays (i.e., QuantiFERON).
**Section** 4 ("Study Vaccines")	**Change/rationale:** Description of study blinding changed. *Candida* antigen control will be blinded by masking the syringes prior to vaccination rather than by modifying the appearance of the antigen.	
	Throughout the Section	Throughout the Section
**Section** 4 ("Study Vaccines")	**Change/rationale:** The final concentration of the MVA85A/AERAS-485 manufactured for the study is stated, and the volume of vaccine (and control) to achieve the planned dose is also specified (i.e., 0.06 ml).	
	Throughout the Section	Throughout the Section
**Section** 4 ("Study Vaccines")	**Change/rationale:** References to the storage conditions for the *Candida* antigen control have been changed to 2-8° C.	
	Throughout the Section	Throughout the Section

Section Number (Title)	Version 2.0	Version 3.0
**Section** 5.1.5 ("Safety Monitoring Committee")	**Change/rationale:** Has been modified to clarify the purpose of disseminating the findings and conclusions of the Safety Monitoring Committees reviews	
	The conclusions of the SMC will be communicated to the investigators and the IRB/Ethical Committees and the national regulatory authority for their concurrence. The sponsor agrees to abide by the decision of its SMC with concurrence of the national regulatory authority, the institutional review board or ethics committee and the principal investigator.	The conclusions of the SMC will be communicated to the investigators and the IRB/Ethical Committees and the national regulatory authority for their information. The sponsor agrees to abide by the decision of its SMC.
Appendix A ("Subject Diary")	**Change/rationale:** Minor modifications were made to Appendix A ("Subject Diary") to improve the clarity of the questions.	
	Throughout the Appendix	Throughout the Appendix
Throughout	**Change/rationale:** Other minor editorial changes and corrections of inconsistencies were also made	

12.3 List of Changes from Protocol Version 3.0 to Version 4.0

The changes that were made to protocol version 3.0 (dated 30 January 2009) to create version 4.0 of the protocol are described below.

Section Number (Title)	Version 3.0	Version 4.0
**Study Abstract**	**Change/rationale:** Although Study Groups 1 and 5 should be large enough to test promising immune response assays for possible correlation with vaccine protection, whenever possible remaining cells and plasma from all groups will also be saved for future immune assays. This will <u>not</u> involve the collection of additional blood.	
	All infants, except for 180 (Study Groups 2-4), will have PBMC collected and stored for future studies of MVA85A/AERAS-485-induced immune correlates of protection…	All infants will have PBMC and plasma collected and stored for future studies of MVA85A/AERAS-485-induced immune correlates of protection…
**Section** 3.5.3 ("Immunology Laboratory Evaluations")	**Change/rationale:** As above	
	Plasma remaining from the immunologic assays in Study Groups 2-4 and from the cells collected in Study Group 1 and 5 will be stored frozen for possible future testing to evaluate immune responses.	Plasma and cells remaining from the immunologic assays in Study Groups 1-5 will be stored frozen for possible future testing to evaluate immune responses.
**Section** 7.4.1 ("Intracellular Cytokine Staining Assay")	**Change/rationale:** Intracellular cytokine staining will be done after stimulation with *Mtb* antigen 85A (Ag85A) only.	
	The primary variables of interest for assessment of immune response to MVA85A/AERAS-485 will be the percentage of CD4 and CD8 T cells that produce any of three cytokines (IFN-γ, TNF-α, and/or IL-2) or a combination of the three cytokines simultaneously following stimulation with peptide pools	The primary variables of interest for assessment of immune response to MVA85A/AERAS-485 will be the percentage of CD4 and CD8 T cells that produce any of three cytokines (IFN-γ, TNF-α, and/or IL-2) or a combination of the three cytokines simultaneously following stimulation with peptide pools

Section Number (Title)	Version 3.0	Version 4.0
	containing the entire amino acid sequence of Ag85A and parts of Ag85B (Ag85A/b), and the complete pool of TB10.4…Descriptive statistics will be used to summarize percentage CD4 and CD8 T cell response by treatment group and stimulation antigen (Ag85A/b and TB10.4), for all subjects in the ICS immunogenicity cohort.	containing the entire amino acid sequence of Ag85A…Descriptive statistics will be used to summarize percentage CD4 and CD8 T cell response by treatment group and stimulation antigen (Ag85A), for all subjects in the ICS immunogenicity cohort.
Throughout	**Change/rationale:** Other minor editorial changes and corrections of inconsistencies were also made	

12.4 List of Changes from Protocol Version 4.0 to Version 5.0

The changes that were made to protocol version 4.0 (dated 29 May 2009) to create version 5.0 of the protocol are described below.

Section Number (Title)	Version 4.0	Version 5.0
**Section 2.1 ("Objectives") and Study Abstract**	**Change/rationale:** A new exploratory sub-study was added to evaluate the innate immune response to vaccination. The sub-study is optional and a separate consent will be obtained	
	Not present	Exploratory objective: To evaluate the innate immune response to MVA85A/AERAS-485 vaccine in a sequentially-enrolled subset of 100 infants using gene expression and transcriptional profile of blood collected at Study Day 1 as analyzed by microarray analysis.
**Section 2.2 ("Study Design") and Study Abstract**	**Change/rationale:** The study may not be limited to a single site if it becomes necessary to increase the enrollment rate.	
	The study is planned at a single site in South Africa.	The study is planned at a single site in South Africa but may be executed at more than one site.
**Section 3.1 ("Summary Schedule of Subject Evaluations") and throughout protocol**	**Change/rationale:** Two follow-up visit days were removed to reduce the strain of frequent early study visits on parents and infants	
	Infants…will be followed up on a regular schedule at Study Day 7 (±2 days), Study Day 14 (±2 days), Study Day 28 (± 5 days), Study Day 56 (± 5 days), Study Day 84 (± 5 days) and every 84 days (± 7 days) until the end of the study.	Infants…will be followed up on a regular schedule at Study Day 7 (±2 days), Study Day 28 (± 5 days), Study Day 84 (± 5 days) and every 84 days (± 7 days) until the end of the study.
**Section 3.1 ("Summary Schedule of Subject Evaluations") and throughout protocol**	**Change/rationale:** See change above related to optional sub-study.	
	Not present	Parents of infants will also be requested to consent to have <1 mL of blood collected by heel prick at Study Day 1; consent will be sought for subjects in any of the five study groups until blood from 100 infants is collected. This

Section Number (Title)	Version 4.0	Version 5.0
		additional blood collection is optional and a separate consent will be required. The blood will be used to evaluate the innate immune response post-vaccination by transcriptional analysis of whole blood.
**Tables 1-5**	**Change/rationale:** Follow-up schedules were modified to reduce the frequency of blood draws and the length and frequency of early follow-up visit	
	Changes include<br>1. Visits on Study Days 14 and 56 removed<br>2. Screening blood draws for Hepatitis B removed due to low prevalence of infant Hepatitis B infection in this cohort<br>3. Injection site photos at Study Days 7 and 28 removed<br>4. Obtaining consent no longer limited to within 84 days prior to randomization (however, parent/guardians willingness to continue on the study will be assessed at each screening visit)<br>5. QuantiFERON test will not be repeated if chemistry/hematology need to be repeated for Study Group 1 infants	
**Section 3.2.1 ("Recruitment and Informed Consent")**	**Change/rationale:** Procedures for identifying infants for the study have been modified to improve study enrollment rates.	
	Not present	Study staff may also approach parent/guardians of infants at home using information obtained from clinic records, birth records, word of mouth referrals from community contacts, or after direct contact parents/guardians.
**Section 3.2.3.1 ("Inclusion Criteria") and throughout the protocol**	**Change/rationale:** Inclusion criterion 1 was changed to avoid losing infants who may have had a late Prevenar injection	
	1. Age of 126 through 154 days on the day of randomization (Study Day 0)	1. Age of 126 through 182 days on the day of randomization (Study Day 0)
**Section 3.2.3.2 ("Exclusion Criteria")**	**Change/rationale:** Exclusion criterion 11 was modified to clarify what was considered a significant exposure to TB	
	11. Shared residence since birth with an individual with tuberculosis or on anti-tuberculosis treatment	11. Shared residence since birth with an individual with active tuberculosis or on anti-tuberculosis treatment for less than 2 months
**Section 3.5.6 ("Diagnosis of Tuberculosis and Case Verification")**	**Change/rationale:** Criteria were added to minimize unnecessary repeat admissions to the CV ward	
	Not present	Asymptomatic children will be admitted to the case verification ward for investigation once for each new cohabiting household tuberculosis contact identified and will be readmitted only if they have continued exposure to an inadequately treated cohabiting household tuberculosis contact. Symptomatic children will be readmitted to the case verification ward if they have new symptoms, or if they were not previously treated for tuberculosis disease and no alternative diagnosis of

Section Number (Title)	Version 4.0	Version 5.0
		their symptoms was made, or if a previous course of anti-tuberculous treatment has since been completed. Children who are receiving prophylactic isoniazid monotherapy may be readmitted to the case verification ward for investigation.
**Section 3.5.6 ("Diagnosis of Tuberculosis and Case Verification")**	**Change/rationale:** The language regarding the provision of TB prophylaxis and treatment was updated to be sure that protocol guidelines would continue to match SA national guidelines. Language was also added to specify that the records of infants diagnosed with TB outside of the clinical site would be reviewed to identify possible study endpoints.	
	The results of all examinations will be summarized and provided to the parent/guardian in a letter. If a diagnosis of TB is made a letter will also be sent to the TB medical officer in the area where the child lives with all test results and a recommendation on treatment. If a diagnosis of TB is made the parent/guardian will be given a one-week supply of anti-TB medication, following treatment guidelines of the tuberculosis control program, and instructed to contact their local clinic.	The results of all examinations will be summarized and provided to the parent/guardian in a letter. If a diagnosis of TB is made or an infant is in close contact with an adult diagnosed with TB (including infants identified during screening), a letter will also be sent to the TB medical officer in the area where the child lives with all test results and a recommendation on treatment. If a diagnosis of TB is made or an infant is in close contact with an adult diagnosed with TB (including infants identified during screening), the parent/guardian will be given a one-week supply of anti-TB medication and instructed to contact their local clinic, which will be responsible for ongoing management according to current South African national TB programme guidelines.<br><br>The hospital records of any children enrolled in the study who may be diagnosed with TB disease as part of routine care in other health facilities will be reviewed to determine if the clinical diagnosis meets the study case definition of TB disease, as defined by the TB Case Definition Endpoints in Section 7.3.1.
**Section 5 ("Safety") and Appendix D ("SAE Reporting Scheme")**	**Change/rationale:** The process flow for managing and reviewing adverse events has been updated throughout the section and the appendix.	
**Section 5.1.5 ("Safety Monitoring Committee")**	**Change/rationale:** A second SMC review for safety was added.	
	Not present	The SMC will conduct a second unblinded-by-group safety and risk review after the 1000th infant completes the Study Day 84 visit.

Section Number (Title)	Version 4.0	Version 5.0
	**Change/rationale:**	
**Section 7 ("Statistical Considerations")**	**Change/rationale:** Modifications were made throughout this section to describe the TB case definition, which was further refined to improve specificity of the endpoint, and to describe how the new TB case definition will be analyzed including definitions of the analysis populations. Also added was a section on the exploratory endpoint for microarray analysis.	
**Section 7.8 ("Safety Reviews")**	**Change/rationale:** A new section was added to the safety reviews by the Safety Monitoring Committee and to describe an additional planned review.	
Throughout	**Change/rationale:** Minor editorial change, and changes to clarify or correct inconsistencies in the protocol were also made	

12.5 List of Changes from Protocol Version 5.0 to Version 6.0

The changes that were made to protocol version 5.0 (dated 9 October 2009) to create version 6.0 of the protocol are described below.

Section Number (Title)	Version 5.0	Version 6.0
**Study Abstract and Section 2.2 ("Study Design")**	**Change/rationale:** Due to the slower than expected rate of enrollment, the study is being amended to follow infants longer to preserve the study's power (see also Section 7.6 change below). Parents will be reconsented for the extended follow-up.	
	The total duration of follow-up is between 537 and 728 days.	Enrollment is expected to be completed in 21 months. Given completion of enrollment in 21 months, all infants will be followed for at least 9 months after the last infant is enrolled into the study. The total duration of follow-up for each infant is at least 9 months and up to 33 months, with additional follow-up time to be determined based on sample size review.
**Study Abstract and Section 2.2 ("Study Design")**	**Change/rationale:** The interval allowed between study vaccination and other vaccinations has been reduced as previous studies have not demonstrated an effect by MVA85A/AERAS-485 on immune response to routine childhood vaccinations.	
	All infants entered into the study will receive a conjugated pneumococcal vaccine (Prevenar®) with the scheduled DPT immunizations Study Day -84 through Study Day -28.	All infants entered into the study will receive a conjugated pneumococcal vaccine (Prevenar®) with the scheduled DPT immunizations Study Day -84 through Study Day -14.
**Section 3.1 ("Summary Schedule of Subject Evaluations"), Tables 1-5**	**Change/rationale:** Collection of pre-vaccination urine samples for possible future Mtb diagnostic tests will be discontinued, because it is felt that a sufficient number of samples have already been collected.	
**Section 3.2.3.1 ("Inclusion Criteria")**	**Change/rationale:** To avoid unnecessarily disqualifying otherwise healthy infants, the weight requirements for eligibility have been modified.	
	Weight: by chart >3rd percentile on Study Day 0	Weight: by chart >3rd percentile on Study Day 0 or, if < 3rd percentile, infant has shown a stable growth pattern

Section Number (Title)	Version 5.0	Version 6.0
**Section 3.2.3.1 (“Inclusion Criteria”)**	**Change/rationale:** As previously stated, the interval between study vaccination and other vaccinations has been reduced as previous studies have not demonstrated an effect by MVA85A/AERAS-485 on immune response to routine childhood vaccinations.	
	Must have received age-appropriate doses of pneumococcal vaccine as recommended by the South African Department of Health but no injection within 28 day prior to Study Day 0	Must have received age-appropriate doses of pneumococcal vaccine as recommended by the South African Department of Health but no injection within 14 day prior to Study Day 0
**Section 3.2.3.2 (“Exclusion Criteria”)**	**Change/rationale:** As above.	
	Received a EPI immunization within 28 days prior to Study Day 0	Received a EPI immunization within 14 days prior to Study Day 0
**Section 3.2.3.3 (“Screening Clinical Assessments and Laboratory Tests”)**	**Change/rationale:** As above	
	All infants will receive a conjugated pneumococcal vaccine with the scheduled DPT immunizations Study Day -84 through Study Day -28. Infants who have not received conjugated pneumococcal vaccine injections at least 28 day prior to prior to Study Day 0 will not be eligible for randomization.	All infants will receive a conjugated pneumococcal vaccine with the scheduled DPT immunizations Study Day -84 through Study Day -14. Infants who have not received conjugated pneumococcal vaccine injections at least 14 day prior to prior to Study Day 0 will not be eligible for randomization.
**Section 3.5.6 (“Diagnosis of Tuberculosis and Case Verification”)**	**Change/rationale:** Collection of urine samples in the case verification ward for possible future Mtb diagnostic tests will be discontinued, because it is felt that a sufficient number of samples have been collected.	
	At some time prior to discharge, urine will be collected and stored for possible future testing with Mtb diagnostic tests.	
**Section 7.6 (“Sample Size Considerations”)**	**Change/rationale:** Due to the slower than expected rate of enrollment, the study is being amended to follow infants longer to preserve the study’s power. In addition, the study’s SMC will routinely monitor the study’s progress in addition to monitoring safety.	
	Given a tuberculosis cumulative incidence of 3% in the control group, 1392 subjects per study group will provide a 90% chance of detecting a 60% difference between the treated and control groups based on a two-sided log rank test at a significance level of 0.05.	Given a tuberculosis cumulative incidence of 3% over 18 months in the control group, 1392 subjects per treatment group (2784 subjects total) would be required to demonstrate positive efficacy when the true efficacy of MVA85A/AERAS-485 is approximately 60%. An estimate of 7.5% of subjects lost to follow-up in each treatment group is assumed over 18 months. Calculations are based on a two-sided log-rank test at a significance level of 0.05. Calculations assume 90% power to detect a 60% reduction in tuberculosis rate between the MVA85A/AERAS-485 and control groups; given an estimated enrollment over 21 months, the last subject to enroll in the study will be followed for a minimum of 9 months to maintain statistical power.<br><br>However, delays in recruitment and/or a lower TB incidence may result in a need to extend follow-up to preserve the power of

Section Number (Title)	Version 5.0	Version 6.0
		the study to detect a treatment group difference. To assess the possible impact of departures from planned subject recruitment rate, total number of TB cases accrued, loss to follow-up, or proportional hazards assumptions, an independent statistician not involved with the conduct or analyses of the trial will perform a sample size review using an interim pooled estimate of the TB event rate and the original treatment effect. It is critical that this interim review occur when sufficient TB events have accrued, to allow for a stable estimate, but before the total cohort has been recruited, due to trial management issues. The initial sample size review is proposed to occur while enrollment is ongoing but when approximately 30% of expected events have accrued, but no later than before 75 percent of the total cohort has been recruited. Details of this review and analysis will be documented in a statistical analysis plan, finalized prior to the review, and in the SMC charter. Timing of subsequent reviews will be determined based on planned completion of recruitment closure after 21 months and enrollment rate.<br><br>These reviews will not result in a statistical penalty as no treatment comparisons will be performed. The monitoring of these parameters and possible courses of action will be detailed in a separate statistical analysis plan and in the SMC Charter. Further reviews and/or unblinding by the SMC to treatment assignments will be performed at the discretion of the SMC if, based on their judgment, additional information is warranted to better assess ongoing event rates to achieve the endpoint of the study.<br><br>The SMC mandate, oversight schedule, and membership will be delineated in the SMC Charter. The statistical analysis plans to be prepared for all analyses will be approved by the SMC. Aeras and study site personnel will remain blinded to treatment assignments and interim analyses by-treatment results throughout the study.

Section Number (Title)	Version 5.0	Version 6.0
Throughout	**Change/rationale:** Other minor editorial changes and corrections of inconsistencies were also made	

12.6 List of Changes from Protocol Version 6.0 to Version 8.0

The changes that were made to protocol version 6.0 (dated 18 May 2010) to create version 8.0 of the protocol are described below. Note that version 7.0 of the protocol was reviewed internally but was neither submitted for regulatory review nor implemented by the clinical site.

Section Number (Title)	Version 6.0	Version 8.0
**Study Abstract and Section 2.2 ("Study Design")**	**Change/rationale:** As described in the protocol (Section 7.6, Sample Size Considerations), an independent statistician performed a sample size review using an interim pooled estimate of the TB event rate. Based on this analysis, it was determined that an additional 6 months of follow-up were required to maintain sufficient power to detect a 60% vaccine efficacy.	
	Given completion of enrollment in 21 months, all infants will be followed for at least 9 months after the last infant is enrolled into the study. The total duration of follow-up for each infant is at least 9 months and up to 33 months, with additional follow-up time to be determined based on sample size review.	Given completion of enrollment in 21 months, all infants will be followed for at least 15 months after the last infant is enrolled into the study. The total duration of follow-up for each infant is at least 15 months and up to 39 months.
**Section 3.1, ("Summary Schedule of Subject Evaluations")**	**Change/rationale:** Follow-up was extended as discussed above.	
	Follow up of all infants will be terminated at the next scheduled study visit after the last enrollee completes 9 months of follow-up (Study Day 252).	Follow up of all infants will be terminated at the next scheduled study visit after the last enrollee completes 15 months of follow-up (Study Day 420).
**Section 7.6, ("Sample Size Considerations")**	**Change/rationale:** Follow-up was extended as discussed above.	
	Calculations assume 90% power to detect a 60% reduction in tuberculosis rate between the MVA85A/AERAS-485 and control groups; given an estimated enrollment over 21 months, the last subject to enroll in the study will be followed for a minimum of 9 months to maintain statistical power.	Calculations assume 90% power to detect a 60% reduction in tuberculosis rate between the MVA85A/AERAS-485 and control groups; given an estimated enrollment over 21 months, the last subject to enroll in the study will be followed for a minimum of 15 months to maintain statistical power.
**Throughout**	**Change/rationale:** The name "Aeras Global TB Vaccine Foundation" was changed to "Aeras" to reflect the sponsor's new name.	

13 REFERENCES

1. Colditz G, Berkey C, Mosteller F, Brewer T, Wilson M, et al. The efficacy of Bacillus Calmette-Guerin vaccination in Newborns and Infants I the prevention of tuberculosis: meta-analysis of the published literature. Pediatrics 1995;96 (1): 29-35.

2. McShane H. Prime-boost immunization strategies for infectious diseases. Curr Opin Mol Ther. 2002a;4(1):23-7.

3. Skeiky YA, Sadoff JC. Advances in tuberculosis vaccine strategies. Nat Rev Microbiol. 2006;4(6):469-76.

4. Wiker HG, Nagai S, et al. A family of cross-reacting proteins secreted by Mycobacterium tuberculosis. Scand J Immunol. 1992;36(2):307-19.

5. Brooks JV, Frank AA, et al. Boosting vaccine for tuberculosis. Infect Immun. 2001;69(4):2714-7.

6. McShane H, Behboudi S, et al. Protective immunity against Mycobacterium tuberculosis induced by dendritic cells pulsed with both CD8(+)- and CD4(+)-T cell epitopes from antigen 85A. Infect Immun. 2002;70(3): 1623-6.

7. Huygen K, Content J, et al. Immunogenicity and protective efficacy of a tuberculosis DNA vaccine. Nat Med. 1996;2(8):893-8.

8. Baldwin SL, D'Souza C, et al. Evaluation of new vaccines in the mouse and guinea pig model of tuberculosis. Infect Immun. 1998;66(6):2951-9.

9. McShane H. Developing an improved vaccine against tuberculosis. Expert Rev Vaccines. 2004;3(3):299-306.

Appendix A: Subject Diary

C-020-485 TB020	Subject Number

TB Vaccine Trial

Brewelskloof Hospital
Haarlem Street
Worcester
6850

Aeras Global TB Vaccine Foundation
South African Tuberculosis Vaccine Initiative
University of Oxford

Please complete all pages of the diary card each evening (including the day of vaccination)

DO NOT HESITATE TO CONTACT US IF YOU HAVE ANY CONCERNS OR QUESTIONS

Office: (023) 346 5400 (between 0800 & 1600)

Out of hours: 076 480 4831

Subject Number

If there is nothing to record write "0" *do not leave blank.*

				Have these happened since the last time you recorded your baby's observations?		Measurement (mm)	
Day after Vaccine	Date	Time (24hr)	Temperature in armpit (ºC)	Pain (circle maximum score)	Warmth (0=No 1=Yes)	Redness	Swelling
0	DD/MMM/YYYY			0 1 2			
1	DD/MMM/YYYY			0 1 2			
2	DD/MMM/YYYY			0 1 2			
3	DD/MMM/YYYY			0 1 2			
4	DD/MMM/YYYY			0 1 2			
5	DD/MMM/YYYY			0 1 2			
6	DD/MMM/YYYY			0 1 2			
7	DD/MMM/YYYY			0 1 2			

Pain Score

0= Baby seems unaware of injection site
1= Baby cries when injection site is touched
2= Baby cries when arm is moved

Warmth

Is the injection site on your baby's arm warm?
0= No
1= Yes

Use the tape measure provided to measure ANY redness (bright pink) or swelling, and try to record everything at approximately the same time each evening.

Measure the diameter (size) of redness and swelling by putting the tape measure through the actual vaccine site.

Record all signs your baby has experienced since the last time you recorded them up to and including the time you take the measurements.

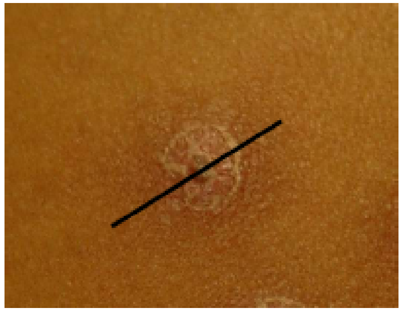

Subject Number

Day	Date	Feeds less than usual	Sleeps less than usual	Sleeps more than usual	Less alert (awake) than usual	Feverish	Irritable (bad tempered)	Diarrhoea (number of times today)	Vomiting (number of times today)
0	DD/MMM/YYYY								
1	DD/MMM/YYYY								
2	DD/MMM/YYYY								
3	DD/MMM/YYYY								
4	DD/MMM/YYYY								
5	DD/MMM/YYYY								
6	DD/MMM/YYYY								
7	DD/MMM/YYYY								

Please indicate if your baby has experienced any of these symptoms since the last time you recorded their symptoms.
Write 'Yes' or 'No' and, if Yes, please also write the number of times.

Subject Number

Other problems	Start date	Stop date	Medications taken (include dose (amount) and how often your baby took them)
	DD/MMM/YYYY	DD/MMM/YYYY	
	DD/MMM/YYYY	DD/MMM/YYYY	
	DD/MMM/YYYY	DD/MMM/YYYY	
	DD/MMM/YYYY	DD/MMM/YYYY	
	DD/MMM/YYYY	DD/MMM/YYYY	
	DD/MMM/YYYY	DD/MMM/YYYY	
	DD/MMM/YYYY	DD/MMM/YYYY	
	DD/MMM/YYYY	DD/MMM/YYYY	
	DD/MMM/YYYY	DD/MMM/YYYY	
	DD/MMM/YYYY	DD/MMM/YYYY	

Please use this table to describe any other symptoms your baby has experienced, even if you do not think they are anything to do with the vaccine, and record any medications your baby takes (remedies, herbal preparations, creams, ointments etc).

Appendix B: Phlebotomy Analysis

In any 24-hour period, phlebotomy for research studies (all studies) in infants and children should not exceed 5% of the total blood volume and ideally should be less than 3% of the total blood volume. In any one-month period, phlebotomy for research studies (all studies) in infants and children should not exceed 10% of the total blood volume.

Per the standard nomogram growth chart, the predicted weight of the youngest trial participants are in Tables A. Standard draws for pediatric Vacutainer® tubes are listed in Table B.

Table A. Allowable Phlebotomy Volumes by Age for Infant Males 10th to < 50th Percentile Predicted Body Weight by age

Age (days)	10th percentile body weight for age in kg	mL Blood Volume per kg	Estimated Blood Volume	Maximum phlebotomy per day	Maximum phlebotomy per 30 days
60	4.0	85	340	10.2	34.0
90	4.8	85	408	12.24	40.8
120	5.4	85	459	13.77	45.9
150	6.0	85	510	15.30	51.0
180	6.8	85	578	17.34	57.8
210	7.2	85	612	18.36	61.2
240	7.6	85	646	19.38	64.6
270	8.0	85	680	20.40	68.0
300	8.2	85	697	20.91	69.7
330	8.6	85	731	21.93	73.1
360	8.8	85	748	22.44	74.8

Table B. Pediatric Phlebotomy Tubes and Their Volumes

Specimen	Volume (mL)	Tube
Chemistry	3 mL	13 x 100 mm x 7.0 ml BD Red Vacutainer®
CBC	3.3 mL	10.25 x 64 mm x 3.3 ml BD Lavender Vacutainer®
CBC	1.2 mL	Microtainer®
QuantiFERON intube	3 mL	
HIV-1	Plasma	10.25 x 64 mm x 3.0 ml BD Red Vacutainer®
Immunology Cells/plasma	8 mL	BD CPT™
Immunology whole blood (optional)	<1 mL	BD Red Microtainer®

Appendix C: Aeras Pediatric Toxicity Table

Note: This table is designed for grading the severity of adverse events in healthy children 3 months to 2 years of age.

Local Site of Injection Symptoms	Grade 1	Grade 2	Grade 3	Grade 4
Injection site pain (pain without touching)	**Minimal or no limitation of use of limb**	**Limitation of use of limb OR greater than minimal interference with usual activities**	**Inability to perform usual activities**	**N/A**
Tenderness (pain when area is touched)	**Minimal or no limitation of use of limb**	**Limitation of use of limb OR greater than minimal interference with usual activities**	**Inability to perform usual activities**	**N/A**
Erythema (Redness)*	**Present, ≤ 1.0 cm in diameter**	**> 1.0 cm to 2.5 cm in diameter**	**>2.5 cm in diameter**	**Local or extensive exfoliative dermatitis**
Induration or Edema	**Present, ≤ 1.0 cm in diameter**	**> 1.0 cm to 2.5 cm in diameter**	**>2.5 cm in diameter**	**Local or extensive exfoliative dermatitis**
Ulceration	**Minimal or no interference with activity**	**Some interference with activity not requiring medical intervention**	**Prevents daily activity and requires medical intervention**	**Requires hospitalization**

* In addition to grading the local reaction at the greatest single diameter, record the measurement as a continuous variable.

Systemic (General)	Grade 1	Grade 2	Grade 3	Grade 4
Decreased oral intake	**Minimal decrease in oral intake**	**Below 50% of normal oral intake in 24 hr**	**No oral intake in 24hr**	**N/A**
Vomiting	**1 episode in 24hr; no interference with activity**	**2-3 episodes in 24 hr OR some interference with activity**	**> 3 episodes in 24 hours OR prevents daily activity**	**N/A**
Diarrhea	**Unformed stool OR 1-3 more stools than baseline in 24 hr**	**Partially liquid stools OR 4-6 more stools than baseline in 24hr**	**Completely liquid stools OR >6 more stools than baseline in 24 hr**	**N/A**
Irritability	**Easily consolable; minimal or no interference with activity**	**Difficult to console; some interference with activity**	**Inconsolable; prevents daily activity**	**N/A**
Fatigue	**Minimal or no interference with activity**	**Some interference with activity**	**Prevents daily activity**	**N/A**
Lethargy	**Minimal decrease in alertness; minimal interference with activity**	**Some interference with activity**	**Unable to achieve normal level of alertness; prevents daily activity**	**N/A**
Illness or clinical adverse event)	**Minimal or no interference with activity**	**Some interference with activity not requiring medical intervention**	**Prevents daily activity and requires medical intervention**	**N/A**

Vital Signs	Grade 1	Grade 2	Grade 3	Grade 4
Fever (Axillary)	**38.0 – 38.4°C<br>100.4 – 101.1°F**	**38.5 - 40°C<br>101.2 - 104°F**	**>40°C<br>>104°F**	**N/A**
Tachycardia – beats per minute<br>3 – 6 months<br>6 months – 1 year<br>1-2 years	**181-200<br>171-190<br>151-170**	**201-220<br>191-210<br>171-190**	**>220<br>>210<br>>190**	**N/A**
Bradycardia – beats per minute<br>3 – 6 months<br>6 months – 1 year<br>1-2 years	**96-105<br>91-100<br>81-90**	**91-95<br>81-90<br>71-80**	**<91<br><81<br><71**	**N/A**
Hypertension (systolic) - mm Hg<br>Females 3 mo – 1 yr<br>Height <25th percentile<br>25th-75th percentile<br>>75th percentile<br>Females 1 yr – 2 yr<br>Height <25th percentile<br>25th-75th percentile<br>>75th percentile<br>Males 3 mo – 1 yr<br>Height <25th percentile<br>25th-75th percentile<br>>75th percentile<br>Males 1 yr – 2 yr<br>Height <25th percentile<br>25th-75th percentile<br>>75th percentile	**98-101<br>100-103<br>103-106<br>99-102<br>102-104<br>104-107<br>95-98<br>98-101<br>102-105<br>99-101<br>102-105<br>105-108**	**>101<br>>103<br>>106<br>>102<br>>104<br>>107<br>>98<br>>101<br>>105<br>>101<br>>105<br>>108**	**Clinical or laboratory evidence of end-organ damage**	**N/A**
Hypertension (diastolic) – mm Hg<br>Females 3 mo – 1 yr<br>Height <25th percentile<br>25th-75th percentile<br>>75th percentile<br>Females 1 yr – 2 yr<br>Height <25th percentile<br>25th-75th percentile<br>>75th percentile<br>Males 3 mo – 1 yr<br>Height <25th percentile<br>25th-75th percentile<br>>75th percentile<br>Males 1 yr – 2 yr<br>Height <25th percentile<br>25th-75th percentile<br>>75th percentile	**53-56<br>54-57<br>56-59<br>57-60<br>58-61<br>60-63<br>51-54<br>53-56<br>55-58<br>55-58<br>57-60<br>59-62**	**>56<br>>57<br>>59<br>>60<br>>61<br>>63<br>>54<br>>56<br>>58<br>>58<br>>60<br>>62**	**Clinical or laboratory evidence of end-organ damage**	**N/A**
Hypotension/<br>Hypovolemia	**N/A**	**Symptomatic, requiring oral fluid replacement**	**Symptomatic, requiring IV fluid replacement**	**N/A**
Tachypnea – breaths per minute<br>3 months – 1year<br>1- 2 years	**39-44<br>31-36**	**45-48<br>37-44**	**>48<br>>44**	**N/A**
Respiratory Distress/<br>Hypoxia	**Wheezing, nasal flaring or retractions; minimal or no interference with activity**	**Some interference with activity or pulse oximetry <95%**	**Prevents normal activity or pulse oximetry <90%**	**N/A**

Serum/Plasma Chemistry	Grade 1	Grade 2	Grade 3	Grade 4
Sodium – hyponatremia<br>mEq/L or mmol/L:	**132 – 134**	**130 – 131**	**<130**	**N/A**
Sodium – hypernatremia<br>mEq/L or mmol/L:	**145- 146**	**147-148**	**>148**	**N/A**
Potassium – hyperkalemia<br>mEq/L or mmol/L<br>3 mo -1 year:<br>1 – 2years:	**5.3 – 5.4**<br>**5.1 – 5.2**	**5.5 – 5.6**<br>**5.3 – 5.4**	**>5.6**<br>**>5.4**	**N/A**
Potassium – hypokalemia<br>mEq/L or mmol/L:	**3.5 – 3.6**	**3.3 – 3.4**	**3.1 – 3.2**	**N/A**
Glucose – hypoglycemia<br>mg/dL:<br>mmol/L:	**55-59**<br>**3.0 – 3.2**	**50-54**<br>**2.8 – 2.9**	**<50**<br>**<2.8**	**N/A**
Glucose – hyperglycemia<br>Fasting - mg/dL:<br>mmol/L:<br>Random – mg/dL:<br>mmol/L:	**101 – 110**<br>**5.7 – 6.0**<br>**110 - 125**<br>**6.1 – 6.8**	**111 – 125**<br>**6.1 – 6.8**<br>**126 - 200**<br>**6.9 – 11.0**	**>125**<br>**>6.8**<br>**>200**<br>**>11.0**	**N/A**
Blood urea nitrogen (BUN) – increased<br>mg/dL:<br>mmol/L:	**21 – 24**<br>**7.5 – 8.9**	**25 – 28**<br>**9.0 – 10.0**	**>28**<br>**>10.0**	**N/A**
Creatinine – increased<br>mg/dL:<br>umol/L:	**0.8 – 0.9**<br>**66 - 82**	**1.0 – 1.2**<br>**83 – 100**	**>1.2**<br>**>100**	**N/A**
Calcium – hypocalcemia<br>mg/dL:<br>mmol/L:	**8.0 – 8.4**<br>**2.00 – 2.10**	**7.5 – 7.9**<br>**1.87 – 1.99**	**<7.5**<br>**<1.87**	**N/A**
Calcium – hypercalcemia<br>mg/dL:<br>mmol/L:	**11.1-11.3**<br>**2.78 – 2.84**	**11.4 – 11.6**<br>**2.85 – 2.92**	**>11.6**<br>**>2.92**	**N/A**
Magnesium – hypomagnesemia<br>mg/dL:<br>mmol/L:	**1.3 – 1.5**<br>**0.52 – 0.62**	**1.1 – 1.2**<br>**0.43 – 0.51**	**<1.1**<br>**<0.43**	**N/A**
Phosphorus – hypophosphatemia<br>mg/dL:<br>mmol/L:	**2.3 – 2.5**<br>**0.73 – 0.80**	**2.0 – 2.2**<br>**0.63 – 0.72**	**<2.0**<br>**<0.63**	**N/A**
Albumin – hypoalbuminemia<br>g/dL:<br>g/L:	**2.5 – 2.7**<br>**25 – 27**	**2.2 – 2.4**<br>**22 – 24**	**<2.2**<br>**<22**	**N/A**
Total protein – hypoproteinemia<br>g/dL:<br>g/L:	**4.4 – 4.6**<br>**44 – 46**	**4.1 – 4.3**<br>**41 – 43**	**<4.1**<br>**<41**	**N/A**
Alkaline phosphatase (ALP)<br>– increased	**1.1 – 2.0 x ULN****	**2.1 – 3.0 x ULN**	**3.1 – 10 x ULN**	**>10 x ULN**
Liver Function Tests (LFT)<br>AST, ALT, GGT – increased	**1.1 – 2.5 x ULN**	**2.6 – 5.0 x ULN**	**5.1 – 10 x ULN**	**>10 x ULN**
Bilirubin (with any increase in LFT)<br>- increased	**1.1 – 1.25 x ULN**	**1.26 – 1.5 x ULN**	**1.51 – 1.75 x ULN**	**>1.75 x ULN**
Bilirubin (with normal LFT)<br>- increased	**1.1 – 1.5 x ULN**	**1.6 – 2.0 x ULN**	**2.1 – 3.0 – x ULN**	**>3.0 x ULN**
Cholesterol – increased<br>mg/dL:<br>mmol/L:	**171 - 185**<br>**5.1 – 5.5**	**186 – 199**<br>**5.6 – 6.0**	**>199**<br>**>6.0**	**N/A**
Pancreatic enzymes<br>amylase, lipase – increased	**1.1 – 1.5 x ULN**	**1.6 – 2.0 x ULN**	**2.1 – 5.0 x ULN**	**>5.0 x ULN**

** ULN is the upper limit of the normal age-appropriate reference range.

Hematology	Grade 1	Grade 2	Grade 3	Grade 4
Hemoglobin (3-6 mo) –<br>g/dL:<br>g/L:<br>Hemoglobin (6mo -2yr)–<br>g/dL:<br>g/L:	 **9.0 – 9.4**<br>**90 – 94**<br><br>**10.0 – 10.4**<br>**100 – 104**	 **8.5 – 8.9**<br>**85 – 89**<br><br>**9.5 – 9.9**<br>**95 - 99**	 **<8.5**<br>**<85**<br><br>**<9.5**<br>**<95**	**N/A**
WBC – increased cells/mm^3	**18,700 – 22,000**	**22,100 – 25,000**	**>25,000**	**N/A**
WBC – decreased cells/mm^3	**4,500 – 5,500**	**3,500 – 4,400**	**<3,500**	**N/A**
Lymphocytes - decreased cells/mm^3	**2,000-2,700**	**1,500-1,900**	**<1,500**	**N/A**
Neutrophils - decreased cells/mm^3	**750-990**	**500-740**	**<500**	**N/A**
Eosinophils – increased cells/mm^3	**850 – 1,500**	**1,501 – 5,000**	**>5,000**	**N/A**
Platelets - decreased cells/mm^3	**125,000 – 140,000**	**100,000 – 124,000**	**<100,000**	**N/A**
Prothrombin Time (PT)/International normalized ratio (INR) – increased	**1.1 – 1.2 x ULN****	**1.3 – 1.4 x ULN**	**>1.4 x ULN**	**N/A**
Partial thromboplastin time (PTT) – increased	**1.1 – 1.2 x ULN**	**1.3 – 1.4 x ULN**	**>1.4 x ULN**	**N/A**
Fibrinogen – increased<br>mg/dL:<br>g/L:	 **400 – 500**<br>**4.00 – 5.00**	 **501 – 600**<br>**5.01 – 6.00**	 **>600**<br>**>6.00**	**N/A**
Fibrinogen – decreased<br>mg/dL:<br>g/L:	 **150 - 170**<br>**1.50 – 1.70**	 **125 – 149**<br>**1.25 – 1.49**	 **<125**<br>**<1.25**	**N/A**

** “ULN” is the upper limit of the normal age-appropriate reference range.

Urine	Grade 1	Grade 2	Grade 3	Grade 4
Protein	**Trace**	**1+**	**2+**	**N/A**
Glucose	**Trace**	**1+**	**2+**	**N/A**
Blood (microscopic) – red blood cells per high power field (rbc/hpf)	**6-10**	**11 – 50**	**>50 and/or gross blood**	**N/A**

Appendix D: SAE Reporting Scheme

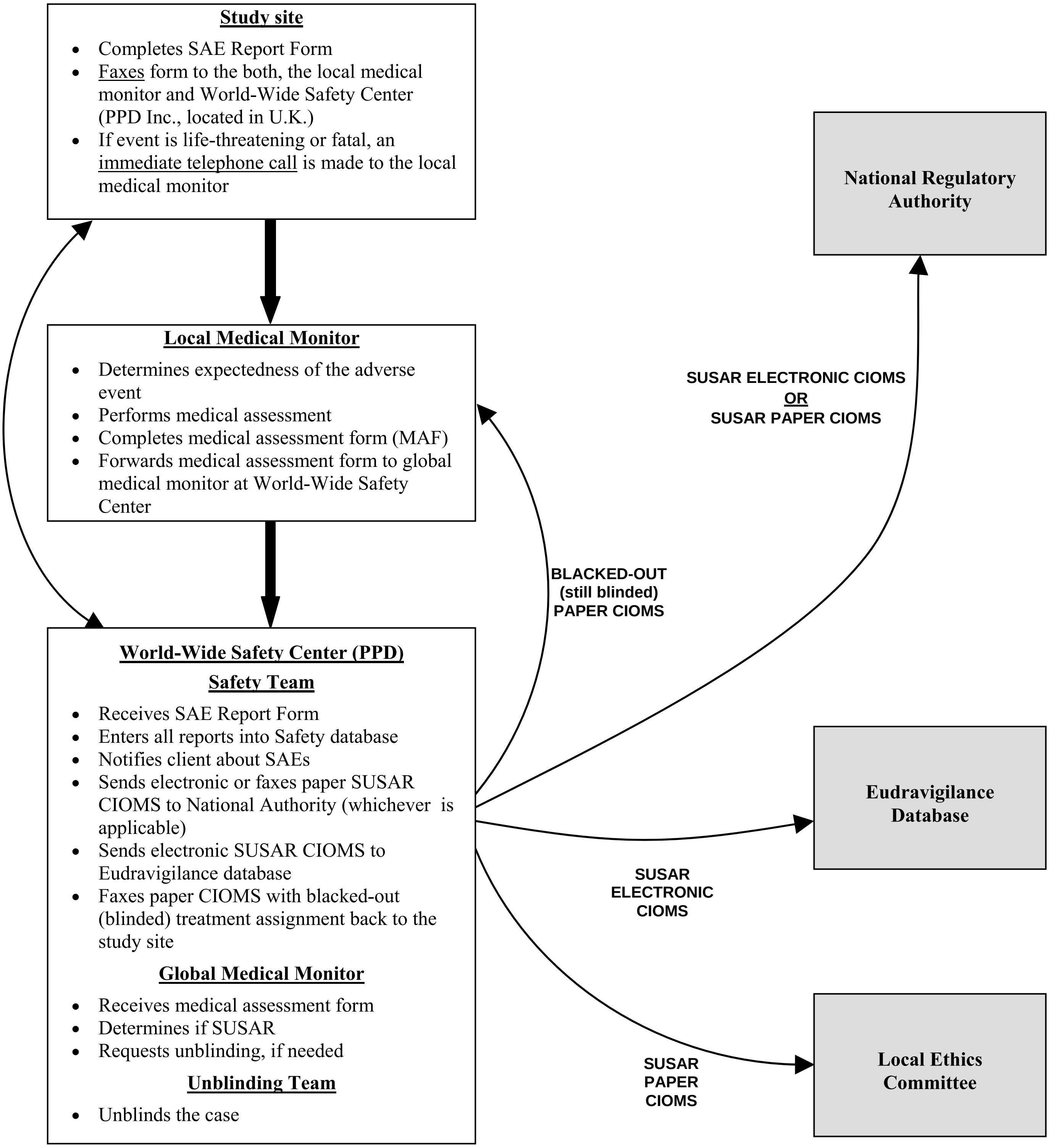

Supplement: S1 Protocol — (PDF) [file pone.0141623.s004.pdf]
