# Supplementary material for: Evaluation of Xpert® MTB/RIF Assay in Induced Sputum and Gastric Lavage Samples from Young Children with Suspected Tuberculosis from the MVA85A TB Vaccine Trial
Source: PLoS One. 2015 Nov 10;10(11):e0141623. doi: 10.1371/journal.pone.0141623 (PMC4640848; doi:10.1371/journal.pone.0141623)
Supplement: S1 Table — (DOCX) [file pone.0141623.s005.docx]

**S1 Table. A profile or account of each sample result from MGIT culture for *Mycobacterium tuberculosis***

|  | **1^st^ gastric lavage** | **2^nd^ gastric lavage** | **1^st^ induced sputum** | **2^nd^ induced sputum** | **TOTAL** |
| --- | --- | --- | --- | --- | --- |
| **Negative result** | 1078 | 1119 | 1090 | 1119 | 4406 |
| **Positive result** | 16 | 16 | 17 | 8 | 57 |
| **Contaminated** | 69 | 27 | 45 | 26 | 167 |
| **Sample not obtained** | 31 | 41 | 40 | 47 | 159 |
| **Mycobacteria other than *Mycobacterium tuberculosis*** | 20 | 11 | 22 | 14 | 67 |
| **TOTAL** | 1214 | 1214 | 1214 | 1214 | 4856 |
