# Supplementary material for: Evaluation of Xpert® MTB/RIF Assay in Induced Sputum and Gastric Lavage Samples from Young Children with Suspected Tuberculosis from the MVA85A TB Vaccine Trial
Source: PLoS One. 2015 Nov 10;10(11):e0141623. doi: 10.1371/journal.pone.0141623 (PMC4640848; doi:10.1371/journal.pone.0141623)
Supplement: S2 Table — (DOCX) [file pone.0141623.s006.docx]

**S2 Table. A profile or account of each sample result from Xpert MTB/RIF for *Mycobacterium tuberculosis***

|  | **1^st^ gastric lavage** | **2^nd^ gastric lavage** | **1^st^ induced sputum** | **2^nd^ induced sputum** | **TOTAL** |
| --- | --- | --- | --- | --- | --- |
| **Negative result** | 1150 | 1140 | 1150 | 1140 | 4580 |
| **Positive result** | 4 | 11 | 7 | 4 | 26 |
| **Invalid** | 13 | 8 | 11 | 15 | 47 |
| **Sample not obtained** | 47 | 55 | 46 | 55 | 203 |
| **TOTAL** | 1214 | 1214 | 1214 | 1214 | 4856 |
